# Exploring the “led” in health professional student-led experiences: a scoping review

**DOI:** 10.1007/s10459-024-10355-x

**Published:** 2024-10-24

**Authors:** Dean Lising, Jodie Copley, Anne Hill, Julia Martyniuk, Freyr Patterson, Teresa Quinlan, Kathryn Parker

**Affiliations:** 1 University Health Network, Toronto, Canada; 2 Department of Physical Therapy, Temerty Faculty of Medicine, University of Toronto, Toronto, Canada; 3 School of Health and Rehabilitation Sciences, The University of Queensland, Brisbane, Australia; 4 University of Toronto, Toronto, Canada

**Keywords:** Student-led, Student-run, Student leadership, Practice, Curriculum, Interprofessional Education

## Abstract

To support a complex health system, students are expected to be competent leaders as well as competent clinicians. Intentional student leadership development is needed in health professional education programs. Student-led experiences such as student-run clinics and interprofessional training wards, are practice-based learning opportunities where learners provide leadership to clinical services and/or address a gap in the system. Given the absence of leadership definitions and concepts, this scoping review explored how student leadership is conceptualized and developed in student-led experiences. The review was conducted in accordance with best practices in scoping review methodology within the scope of relevant practice-based student-led experiences for health professional students. The research team screened 4659 abstracts, identified 315 articles for full-text review and selected 75 articles for data extraction and analysis. A thematic analysis produced themes related to leadership concepts/theories/models, objectives, facilitation/supervision, assessment and evaluation of curriculum. While responding to system gaps within health professional care, student-led experiences need to align explicit leadership theory/concepts/models with curricular objectives, pedagogy, and assessments to support health professional education. To support future student-led experiences, authors mapped five leadership student role profiles that were associated with student-led models and could be constructively aligned with theory and concepts. In addition to leveraging a student workforce to address system needs, student-led experiences must also be a force for learning through a reciprocal model of leadership and service to develop future health professionals and leaders.

**Supplementary Information:**

The online version contains supplementary material available at 10.1007/s10459-024-10355-x.

## Introduction

Health professional leadership is widely recognized to be necessary for an effective healthcare system (World Health Organization, 2020). Leadership has profound impacts on all aspects of organizational culture in health care and influences performance, retention, and sustainability (Johnson et al., 2021; Swanson et al., 2010). Leadership is a part of all health professionals’ roles and involves leading people, care plans, projects and programs to ensure optimal outcomes for patients, caregivers and communities (Johnson et al., 2021); Kruk et al., 2018; . Contemporary leadership models include health system leadership frameworks and professional competencies in health professional education (HPE). A common framework with health leaders include the LEADS (Leads self, Engages others, Achieve results, Develop coalitions, System transformations) in a Caring Environment capabilities framework which represents skills, behaviors, abilities, and knowledge required to lead in different sectors of the health system (Canadian College of Health Leaders, 2024). Collective and collaborative leadership frameworks involve multiple professionals sharing viewpoints and knowledge with the potential to influence the quality of care and staff well‐being. Collective leadership models and interventions have been shown in Cochrane systematic reviews (Silva et al., 2022) to be an important influence on professional practice, healthcare outcomes, staff well‐being and leadership outcomes. Professional competency profiles include leadership as a key domain of professional (Frank et al., 2015) or collaborative practice (Canadian Interprofessional Health Collaborative (CIHC), 2024). All these models stipulate that leaders need to effectively practice leadership tasks (manage others, develop partnerships, achieve results, etc.) while navigating the complexities of the healthcare system. As such, students are expected to learn to be competent leaders as well as competent clinicians in a complex, changing health care system.

Intentional student leadership development within HPE curriculum prepares graduates to successfully lead in the health system (Clarke, 2015; Diggele et al., 2020). However, leadership development within HPE curricula is still wanting (Frich et al., 2015; Sadowski et al., 2018). Emerging concepts such as adaptive complex systems, transformative system change and social justice are only beginning to be integrated in medical and health professional competency frameworks and curricula (Thoma et al., 2023, CIHC, 2024). This is especially true for the concept of collaborative leadership where little or no undergraduate curricula exist to enable future healthcare leaders as collaborative, compassionate leaders of meaningful change (MacPhee et al., 2013). Furthermore, the need for experiential learning opportunities, that are contextually relevant over more traditional didactic teachings, is critical to effective leadership development (Swanick & McKimm, 2012; Warren & Carnall, 2011, Sultan et al., 2019) In response to this need, universities and practice organizations are integrating leadership development in HPE programs; however, this development remains variable in the content, delivery and level of targeted students (Careau et al., 2014; Sonnino, 2016). Student-led services and clinics are emerging within education and clinical practice as an avenue to not only meet gaps in service but also facilitate opportunities for leadership in ‘real world’ or clinical environments for students.

This paper explores the concept of leadership in student-led experiences (SLEs). For the purposes of this paper, SLEs are defined as unique practice-based learning opportunities where health professional learners, as part of their curriculum placement requirements, learn and provide leadership in work-based activities to support new or existing service delivery that addresses an identified gap in the workplace. There is growing recognition of the value of student leadership through real-life experiential learning, with increasing student-led activities integrated into requisite practice-based curricula (Ahern and O’Donnell, 2022). Through required practice curricula activities, students learn leadership by ‘doing’ and apply knowledge through context-based learning in the authentic roles and environments of their future profession (Koens et al., 2005; Schutte et al., 2015). Practice-based student-led activities offer an opportunity to develop student leadership while also providing a service that may not otherwise be available for patients, caregivers, and communities. To support the development of clarity in the HPE field regarding how leadership is conceptualized, taught and experienced by students, leadership as a concept and development model in SLEs needs to be explored further.

The focus of the SLE literature has been on descriptions of interventions, delivery models, student experiences and service outcomes, but the leadership aspect of the student experience and how this leadership is developed is not clear. Among the literature reviews published to date on SLEs, they have been most commonly described as student-run clinics and interprofessional training wards (Briggs & Fronek, 2020; Hopkins et al., 2022; Oosterom et al., 2019; Schutte et al., 2015; Suen et al., 2020; Tokolahi et al., 2021; Wynne & Cooper, 2021). According to these SLE models, students may be responsible for clinical interactions and for organizational aspects of service delivery (Brewer et al., 2016; Wynne & Cooper, 2021). Within the literature, the operations of clinics and wards tend to be well described, but a scan of the extant literature suggests that common elements such as leadership objectives, theory and pedagogy remain underreported. That is, the SLE literature tends to report on operational implementation rather than the elements of the experiences that support student leadership development. This gap indicates the need to clarify what type of student leadership is being developed and how to ensure that future student-led activities achieve outcomes of developing leadership in health professional students. In turn, we can ensure that best practices in student leadership development are adequately understood, implemented and evaluated.

A number of literature reviews have explored student leadership in health professional and interprofessional education (IPE) programs (Brewer et al., 2016; Careau et al., 2014; Hoffman et al., 2008; Scammell et al., 2020). Many of these have focused on leadership theory, pedagogy and content in university-based courses situated on campus rather than practice-based activities situated in clinical settings. A scoping review by Scammel (2019) recognized the variability of pedagogical methods and the integration of student leadership in university-based curricula specific to undergraduate nursing education. Brewer et al. (2016) noted a gap in leadership definitions, theories and concepts in both pre licensure curricula and post licensure practices in IPE rather than HPE experiences.

In contrast, a review by Nagel et al. (2022) recognized the gap in the definition and conceptualization of leadership in student-run health initiatives in community-based practice based activities. They defined these initiatives as student-infused, student-run and student-led depending on the level of student versus academic institution initiation and operations. This review focused on the level of independence students experienced while providing a service; however, insight into the conceptualization and development of leadership competence was not explored. Through the reviews of current literature, we found few explorations of leadership development in practice settings.

In student-run clinics, wards, and community initiatives, there is more of a focus on “what” SLEs are rather than “how” student leadership is occurring and being learned in SLEs. Given that students are asked to lead or run clinics, wards, and projects, it is implied that they will develop leadership skills but it is not explicit as to how this will be facilitated. However, the idea of SLEs may seem enticing to students and faculty as a means of building leadership capacity. Beneath the surface of providing student-led services, are we providing enough depth and structure in the leadership curriculum for students to develop their leadership skills? These are the key tensions and gaps we hope to explore in this scoping review. Without adequately addressing leadership development in these services, we may be addressing workplace gaps but underutilizing the practice environment to truly prepare our future health care leaders. If SLEs are not filling the gap in leadership development, newly graduated health professionals may not be ready for leadership roles in the context of escalating demand for care and decreasing the supply and retention of health care staff (Al-Dossary et al., 2016; Hatlevik, 2012; Scammel et al., 2020). The aim of this scoping review was to understand what is known about leadership concepts in the literature on SLEs and how these concepts are developed in practice-based curricula. Our primary research question was as follows: *How is student leadership conceptualized and developed in practice-based student-led experiences?*

## Methodology

This study utilized a scoping review to examine knowledge from across-study designs on practice-based student-led experiences in the HPE literature. Scoping reviews are a methodology for mapping areas of research and presenting results in an accessible format (Arksey & O’Malley, 2005; Levac et al., 2010). Our goal was not to appraise the quality of the literature but rather to create an overview of the available knowledge about how leadership is defined, conceptualized and developed through a review of a wide range of SLE literature.

This review was conducted in accordance with the scoping review methods outlined by Arksey and O’Malley (2005), advanced by Levac et al. (2010) and updated in further scoping review guidelines (Peters et al., 2020; Thomas et al., 2017, 2020; Westphaln et al., 2021). The study protocol was registered and updated as per the methodology below (Lising et al., 2022) and accessible via the following: 10.17605/OSF.IO/XH5A2. The use of the scoping review framework and protocol registration promote transparency and replicability of the study findings (Peters et al., 2020). The PRISMA extension for scoping reviews check-list (PRISMA ScR) was completed and is included in the supplemental material.

The research team used a dynamic and reflexive approach throughout our research process to refine and update our methods as needed. Our context as a research team includes SLE leaders (DL, JC, AH, KP, FP, TQ), student-led clinic managers (TQs) and SLE developers (DL, JC, AH, KP, FP, TQ) who co-led the development of the research questions, inclusion/exclusion criteria, and narrative review to ensure the relevance of the study to present-day practice/education models and the dissemination of the study results. Given our role in SLE development, we were interested in SLE models, but as HPE faculty, we were particularly interested in how leadership is integrated into curriculum structures and student learning. Members of the team also have past and present roles in undergraduate and postgraduate HPE curriculum development (AH, DL, JC, FP, and TQ), IPE curriculum development (DL, KP, AH, JC, and TQ) and PhD studies in HPE research (DL), health leadership (DL health administration, quality improvement, KP collaborative change leadership); thus, they were invested in exploring all types of SLE literature. The team’s health professional backgrounds are inclusive of physical therapy (DL), occupational therapy (JC, FP, TQ) and speech pathology (AH). A university information specialist and health science librarian (JM) was also a member of our team to support the study development in accordance with the scoping review methodology, search strategy creation and implementation, and manuscript authorship related to methodology and search. This librarian role is recommended in current scoping review guidelines (Thomas et al., 2017) to ensure that the review is conducted and documented rigorously in adherence with the research question.

### Stage 1: Identifying the research question

A preliminary search of Google Scholar, PROSPERO, and the University of Toronto Library Catalog was conducted to ensure that there were no current or ongoing systematic reviews, scoping reviews or protocols on our research question. The identified concepts in this area of study provided direction and clarity in this initial search and informed the subsequent search processes. Research team members (DL, JC, AH, KP, FP, and TQ) identified the preceding research question to guide the scoping review.

### Stage 2: Identifying relevant studies

Peer-reviewed literature was identified using a comprehensive search strategy developed by JM in collaboration with the rest of the research team. The search strategy was validated through the retrieval of a key set of identified relevant studies. To minimize search errors and enhance the comprehensiveness of the search, the search strategy was peer reviewed following the Peer Review of Electronic Search Strategies (PRESS) guidelines (McGowan et al., 2016). Subject headings and text words related to the following concepts were included in the search: Student Led, Clinical Placements and Leadership. The search strategy was developed and finalized in OVID Medline and then translated to OVID Embase, OVID PsychINFO, OVID AMED, EBSCO CINAHL, EBSCO SportDiscus, and the Cochrane Library. The complete search strategies have been uploaded into the Data Repository Dataverse 10.5683/SP3/UIAG2V (Martyniuk, 2023). No language or date limits were applied to the search. The reason for this is the English filters are not 100% accurate, and most abstracts are translated into English. If a paper passed the title and abstract screening but was excluded during full-text screening due to it being not in English, then it would be marked as not passing full-text due to not being in English. The search results were exported into EndNote to deduplicate records and then imported into Covidence for screening and data extraction. The Preferred Reporting Items for Systematic Reviews and Meta-Analyses (PRISMA) Search extension checklist (Rethlefsen et al., 2021) was used to perform the literature searches. The study search was run on February 14, 2022, and then rerun on May 12, 2023, to ensure that no recently published articles were missed. Relevant gray literature was identified by manually searching Open Grey, Trip Pro, and Google on July 18, 2023. The gray literature search and parameters were also uploaded into Dataverse: 10.5683/SP3/UIAG2V. The gray literature documents found were integrated into Covidence to be selected and screened as per the processes below. Additional papers were identified from the reference lists of the included studies to identify studies that may have been missed within the search.

### Stage 3: Study selection

We included peer-reviewed and grey literature, including quantitative, qualitative, mixed and multimethod research and both comparative (e.g., randomized, controlled, cohort, quasi-experimental) and noncomparative (e.g., survey, narrative, audit) methods. Educational reports and program descriptions related to the topic of SLEs were also included.

Our inclusion and exclusion criteria are listed in Table 1.

**Table 1:** Inclusion and exclusion criteria

Inclusion	Exclusion
English language, empirical and descriptive studies on SLEs including activities where students lead, run, manage, facilitate, direct or deliver service	Conference abstracts or dissertation papers, editorial letters, opinion, critique or commentary articles
SLE leadership activities that are integrated and assessed as part of practice placement/fieldwork/work integrated learning components of a health professional program in a workplace setting where students work to deliver or extend a health service	Solely classroom, project-based or simulation-based activities that are not practice-based (i.e., in a workplace setting) Volunteer experiences that are not integrated and assessed as part of curriculum requirements
SLE activities that are relevant to a health professional’s role in practice	SLE activities that are not relevant to a health professional’s role in practice
Health professions are included as defined by Healthforce Ontario and Queensland health professional listings (Healthforce Ontario, 2022; Office of Queensland, 2022)	Any roles not listed as health professions

Although many SLEs included volunteer student-run clinics, we limited our focus to papers and reviews that related to SLEs embedded into health professional practice-based curricula as a mandatory component to ensure alignment with our context of interest. Voluntary SLEs presuppose preexisting student interest in an area of study and may also have a novel effect on learning (Braun et al., 2019). An integrated practice-based curriculum provides an opportunity for formal assessment of students’ practice competence and skills as well as their readiness for the workplace (Ahern and O’Donnell, 2022), which is not present for volunteer experiences. Considering our authorship team’s perspectives and positionality as curricular faculty, we were interested in experiential learning that engaged the broad student cohort in the required integrated curriculum. However, as many studies and reviews were not transparent or explicit about how their SLE experience was integrated and assessed in practice and/or according to curriculum requirements, studies were included when they were in doubt. Additionally, we included SLE activities that were required by the curriculum as part of a menu of elective options.

**Fig. 1:**
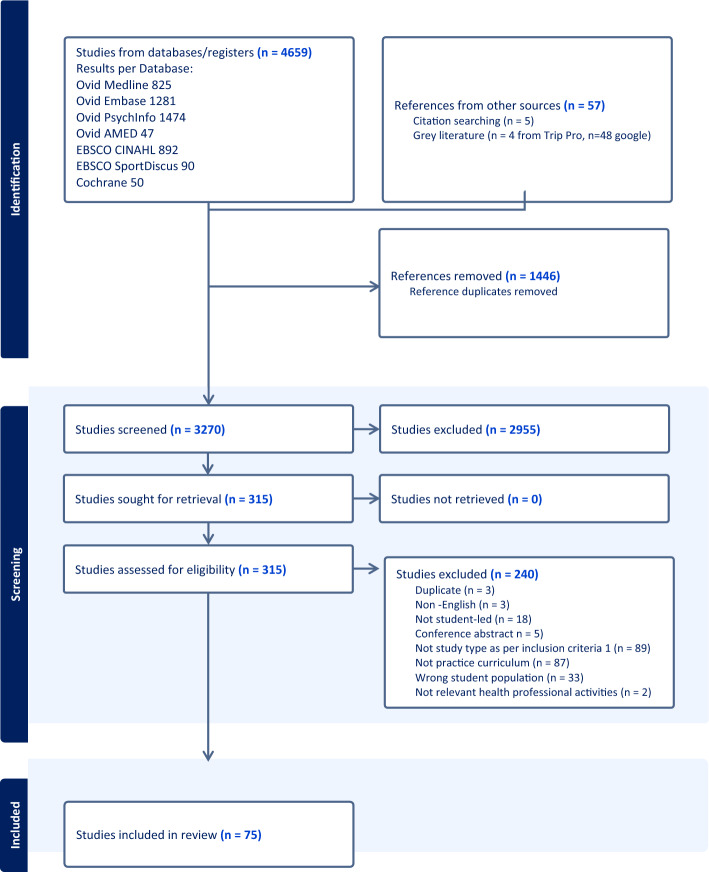
PRISMA flow diagram

The screening questions were developed and pilot tested before we began the full study selection process to confirm a consensus > 75% on the inclusion/exclusion criteria. This process was undertaken by all the research team members (DL, JC, AH, KP, FP, TQ, and JM), with each person testing from a random sample of 100 articles in total. Titles and abstracts were independently reviewed by two members of the team (DL, JC, AH, KP, FP, and TQ) against the eligibility criteria and marked as ‘include’, ‘exclude’ or ‘maybe’. Discrepancies were resolved by discussion and consensus between the pair of reviewers and with a third reviewer when no resolution was reached. Following the completion of the title and abstract screening, full-text articles were retrieved for studies deemed ‘included’ or ‘maybe’, and another pilot test was performed for the full-text screening to ensure consistency. The full texts of the remaining articles were subsequently screened by pairs of reviewers against the eligibility criteria. Conflicts for the full-text screening were resolved by discussion between the pair of reviewers or by a third reviewer. The study selection process was reported using the PRISMA flow diagram in Fig. 1, including reasons for excluding full-text articles.

Through this process, the research team met regularly to review the clarity of the inclusion/exclusion criteria and decision-making processes, ensuring a reflexive and iterative approach. Clarity of the criteria and exclusion tag wording was necessary to ensure standardization of the process given the multiple researchers involved, and regular meetings were held to ensure that all the articles were screened in the same way and that conflicts were resolved through rigorous decision making. The wording of the inclusion and exclusion criteria was updated in the final research protocol and publication. More specifically, we removed “administered” as a leadership synonym and “role-emerging placements” which we determined were not clear or relevant terminology as we prepared to implement our protocol.

### Stage 4: Charting the data

All included studies were reviewed and charted independently by reviewers (DL, JC, AH, KP, FP, and TQ). We used a data extraction form created for this study by our team and pilot-tested using 5 papers together. Final data was reviewed, checked for consistency and completed by DL. Charting is a technique for organizing and interpreting data by sifting, categorizing and sorting material according to key issues and themes (Arksey & O’Malley, 2005; Levac et al., 2010; Thomas et al., 2017).

### Stage 5: Collating, summarizing and reporting the results

To present an overview of all the information retrieved and to establish the extent and nature of the literature, the results of the review were presented using two strategies recommended for scoping reviews (Arksey & O’Malley, 2005; Levac et al., 2010; Thomas et al., 2017):

- 1)A basic numerical overview of the number, type and distribution of included studies and.
- 2)A thematic analysis of relevant study data describing how the results were related to the objectives and questions of the scoping review.

Overall, we created a diagrammatic and tabular format for the included studies, listing the relevant categories above and synthesizing the results via thematic analysis.

In synthesizing the data, each team member (except for JM) brought preexisting knowledge of and experience with SLEs and leadership education. The data were therefore filtered through the team members’ existing expertise (Brannick & Coghlan, 2007; Guba & Lincoln, 1994) and through our subjective interpretation of what is known about this topic (Thomas et al., 2020).

Through this process, we provided an interpretative analysis of the included studies, aligning the development of themes with the aims and questions of the scoping review. We applied descriptive qualitative coding in alignment with our rationale to identify and clarify SLE concepts and definitions within the HPE field as well as to identify key development characteristics related to leadership (Peters et al., 2020). Thematic analysis was conducted through collective team meetings with key themes and conclusions were determined through consensus. The team adopted a subjectivist epistemology (Thomas et al., 2020) given the wide breadth of data, heterogeneous settings, methodologies and ambiguity of the leadership concepts described in the studies. Our thematic coding process included team members familiarizing ourselves with the data, generating initial codes, collating/reviewing codes into potential themes and finally defining/naming themes through consensus meetings included the entire research team (Braun & Clarke, 2006).

### Stage 6: Consultation to provide opportunities for consumer and stakeholder involvement to suggest additional references and provide insights beyond those in the literature

In addition to our team of SLE leaders and managers, we sought additional views within our professional networks to ensure that the study findings would meet the needs of health care professionals and policymakers and to spearhead end-of-study translation. These collaborators were asked to offer their perspectives throughout the study and suggest additional sources of information. Consultation with SLE developers, facilitators, students, HPE researchers and faculty occurred during the study design, and discussion of the preliminary findings was continued to validate the results and to inform future research. We also shared preliminary findings with health care professionals and researchers outside the study team to validate the results and support knowledge translation efforts.

### Research board ethics

Ethics approval is not needed for this scoping review.

## Results

The results are reported through a thematic analysis of relevant study data related to the aims and questions of our scoping review, alongside a diagrammatic and tabular presentation of the included studies.

### Composition of the literature

A total of 75 studies met our scoping review study criteria and were included in the full-text review and extraction. Over 70% of the articles were published between 2013 and 2023, suggesting increased interest in SLEs during the past decade. Thirty-four papers were reports/program descriptions, 16 were mixed/multi-methods, 11 were qualitative, eight were synthesis/reviews and six were quantitative. Forty-two SLEs were uniprofessional, primarily medicine, nursing, OT, PT, or pharmacy, and 33 were interprofessional SLEs with multiple professions.

**Fig. 2:**
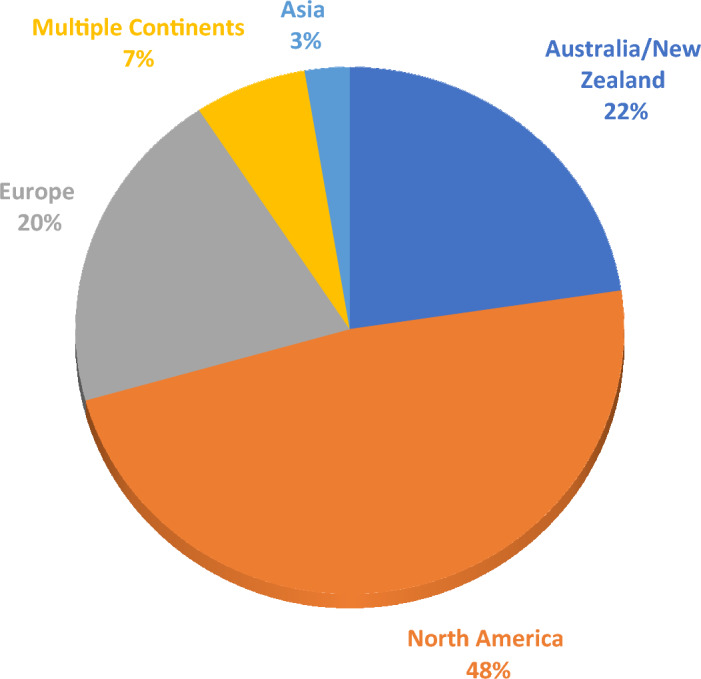
Geographical Distribution of SLEs

Figure 2 summarizes the geography and continental distribution of SLEs.

Further details around composition/activities of SLEs is listed in Table 1 in the supplemental materials.

### SLE terminology, definitions and student activities

Across the included papers, SLEs varied in both terminology and definition. Several common terms were used across the papers, including variations on student-run clinics (n = 21), interprofessional training wards (n = 15) and service learning programs (n = 23). Although most had an operational definition of the service (i.e., clinic, ward, or community service), only 13 of 75 papers explicitly suggested or mentioned leadership development in their definition.

The term “student-run clinic” was used to denote the day-to-day running of primary, community or outpatient clinics, with direct engagement with patients and caseload management (Ahern & O’Donnell, 2022; Wilson et al., 2023). Other similar terminology included student-led clinics and student-run pro-bono clinics for non or underinsured patients (Ng & Hu, 2017; Palombaro et al., 2011; Paparella-Pitzel et al., 2021; Rupert et al., 2022). The terminology “student-assisted” (Frakes et al., 2014) or “student-implemented services” (Barker et al., 2022) was also used, suggesting a less autonomous role for students in these SLEs. Student-led health services (O’Brien et al., 2015) or groups (Patterson et al., 2017) were similar to student-run clinics in providing an operational service within a hospital.

Interprofessional training wards (IPTWs) tended to be defined as the interprofessional collaboration of multiple health professional students situated in the same hospital ward or unit setting of the team. In one definition, IPTWs were explicitly defined as an SLE model in which “students/trainees from different health professions worked together in interprofessional (IP) teams in order to manage the full responsibility for the care of real-life patients in an inpatient-hospital setting while learning interprofessional competencies” (Mihaljevic et al., 2018, p. 2). Interprofessional terminology was also used for similar student-led collaborative models, including IPE placements (Hood et al., 2014) and an interprofessional teaching and learning unit (Braun et al., 2019) including professions such as medicine, nursing, PT and OT. We also noted training ward terminology was used for uniprofessional ward or student teams involving one profession as well.

Service learning programs involving SLE initiatives were also present in the literature. These studies were more explicit with common terminology and definitions of service learning but less so related to service leadership development among students. Most frequently, the definitions included the terms ‘community service’, ‘community engagement’ and ‘civic’ or ‘social’ responsibility (Foli et al., 2014; Groh et al., 2011; Gupta, 2006; Khan & Jacob, 2015; Musolino & Feehan, 2004; Peterson & Schaffer, 1999; Richards et al., 2009; Schneider et al., 2018; Voss, 2016). In most of the definitions, the authors noted a reciprocity in definition between learning and service to the community. For example, service learning was described as a model of both education and community development that aimed to engage students with communities in ways that enhanced their academic experiences while simultaneously serving the needs of the community (Schneider et al., 2018).

**Fig. 3:**
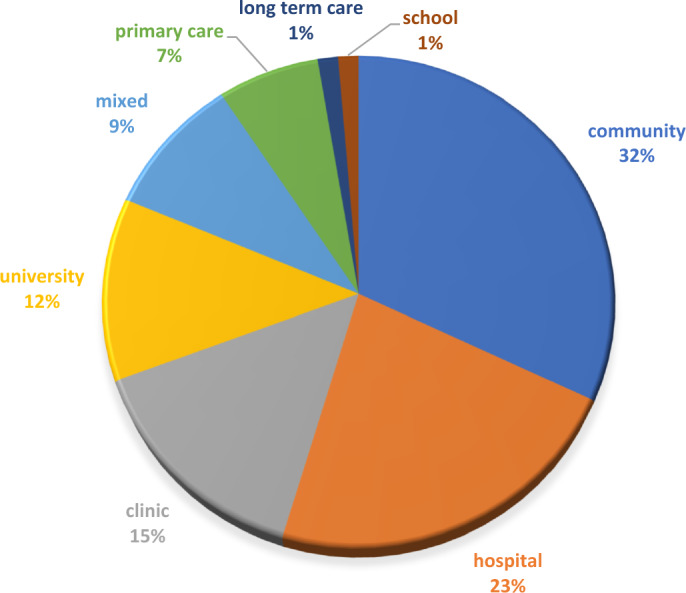
Settings of SLE activities

Figure 3 demonstrates the various sectors and settings where SLE activities have occurred.

It would appear that the majority of SLE activities supported or augmented existing clinical services, predominantly in areas of community, acute, rehabilitation, ambulatory/outpatient and primary care. SLE activities also involved students engaging in health promotion/prevention initiatives, outreach/advocacy and quality improvement projects, particularly in service learning models. Service provision gaps, when described, were most often related to underserved, underinsured or at-risk populations and those marginalized in the community; in the case of service learning projects, health promotion projects such as drug or smoking prevention programs (Childs et al., 2003; Powers et al., 2008), domestic violence (Peterson & Shaffer, 1999) and homelessness were often involved (Ng & Hu, 2017). Fifteen of the studies reviewed described SLEs attending to gaps of service in an inpatient environment, and these were primarily associated with training wards. A minority of the papers described SLEs where the need for additional services for an already defined and serviced diagnostic group was identified prior to the SLE and then provided by students, for example, additional cognitive, upper limb and meal preparation groups in a hospital-based occupational therapy brain injury service (Patterson et al., 2017) or inpatient nutrition management and malnutrition prevention in an internal medicine ward (Braun et al., 2019). In some of the SLE models that were created in response to the pandemic, key drivers of SLEs were not only related to service given to particular patient populations but also related to a reduction in clinical placement opportunities due to isolation policies, closures and staff burnout (Grilo et al., 2020).

**Fig. 4:**
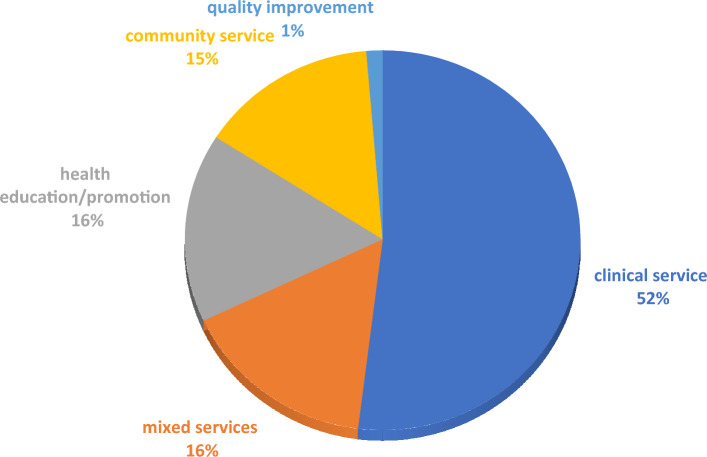
Distribution of SLE activities

Figure 4 demonstrates the various types of activities that students have implemented in SLEs.

Further details around activities of SLEs is listed in Table 1 in the supplemental materials.

## Themes

The resulting themes were interpreted and synthesized from our thematic analysis.

### The boundaries of leadership within SLE theories and concepts

In the included papers, we noted a lack of explicit concepts, theories and frameworks that defined and bounded student leadership. Given our research question, we reviewed studies for the presence of underpinning theories or theoretical or conceptual frameworks that reflected the context of leadership and/or the student-led activity. Theories, concepts and frameworks are the foundations of the paradigms and methods that educators use to teach and share knowledge based on educational science (Varpio et al., 2020). However, in our reporting, we did not differentiate between these terms because it was difficult to infer and categorize given the limited detail provided in most papers.

Of the studies reviewed, only 17 of 75 explicitly described the concept of leadership through an existing theory or theoretical or conceptual framework. We noted several common categories of theoretical concepts underpinning SLEs, ranging from more general knowledge-based theory or learning theories to leadership-specific theories such as clinical leadership/management, interprofessional competencies, servant and transformational leadership and cultural competence.

In their exploratory perspective paper on student-led clinics, O’Brien et al. (2015) noted little literature on the underpinning philosophies and theoretical models of such clinics. The authors proposed the concepts and theories related to communities of practice (Wenger, 1998) as a foundation for the clinic model but adapted this to their context as a community of clinical practice (CoCP). They described their CoCP as a health educational model in a clinical setting with routine yet shifting interactions of students and patients through the community. This model recognized the patient’s part in health education as they bring their expertise and experience to contribute to the students’ learning. A community of practice was also described by Tokolahi et al. (2021) and linked to the underlying learning model of student-led clinics. Neither paper noted a link between communities of practice or other theories as underpinning theory or models for leadership development.

Clinical leadership concepts/management competence was more common in nursing-focused SLEs with hierarchical roles and structures. Within these SLEs, activities related to coordinating and managing care tasks were described (such as the charge nurse role) (Jack et al., 2022; Reime et al., 2022). Clinical leadership, as defined by attributes from a concept analysis (Jack et al., 2022), included effective interpersonal communication skills, contemporary clinical knowledge and being a role model for others. In contrast to task-focused clinical skills, clinical leadership in nursing highlights the importance of interpersonal communication leadership skills (Reime et al., 2022). New nurses in particular were targeted within SLEs to develop clinical leadership, recognizing that often students follow/observe rather than lead/do. Other examples of leadership behaviors underpinning nursing SLEs included an inventory of leadership practices (Foli et al., 2014).

A range of interprofessional competencies and frameworks (Brewer & Stewart-Wynne, 2013; Heath et al., 2019; Lachmann et al., 2013; Mihaljevic et al., 2022, Mink et al., 2021; Morphet et al., 2014) were used as foundational concepts underpinning teamwork in interprofessional SLEs and IPTWs. Various IPE competency frameworks, most commonly the Interprofessional Education Collaborative (IPEC) Competencies (IPEC, 2016) and the Interprofessional Capability Framework (Brewer & Stewart-Wynne, 2013), were cited, but none of those frameworks explicitly acknowledged team leadership competencies. The Canadian Interprofessional Health Collaborative National Interprofessional Competency Framework (Beckman et al., 2022; CIHC, 2024), was also used but the collaborative leadership domain was not identified. Morphet et al., (2014), recognized leadership as part of interprofessional competency standards for medicine and nursing. In another study, CanMEDS (2015), primarily a medical competency framework but also used by other professions, was listed as a  foundational conceptual and theoretical framework aligned with leadership and learning to their IPTW.

In contrast to larger interprofessional groups, Donahue et al. (2001) identified co-leadership principles for pairs of students working as dyad co-leaders. They also highlighted the contingency theory of situational leadership style, which suggests that leadership style is dependent on the task and the level of group ability and supports students in intersecting/selecting leadership models based on their leadership styles.

Although not often present in their definitions, descriptions of service learning SLEs included some explicit reference to the theoretical and conceptual underpinnings of leadership. These articles employed the notion of servant leadership to inform their SLE design. Servant leadership (Goldstein et al., 2009; Groh et al., 2011) occurs when leadership has social responsibility and is in service to stakeholders and the community. Servant leadership has been defined as the principle that one wants to serve first (Greenleaf, 1970). Another key concept linked to service learning was transformative leadership (Foli et al., 2014; Goldstein et al., 2009; Gupta, 2006; Pardo et al., 2022). There were varied descriptions of transformative leadership linked to social change, justice and critical reflection. In some papers, transformative leadership referred to individual growth supported by preceptor relationships (Foli et al., 2014); in others, transformative leadership was related to transforming environments rather than individuals (Goldstein et al., 2009). Gupta (2006) described transformation for societal change on a deeper level by analyzing system injustices and a commitment beyond charity to social change and justice for transformation and transformative learning.

As many of the SLEs described involved students working with underserved marginalized populations, they also included foundational concepts of cultural competence and cultural pluralism as part of course theory (Barker et al., 2022; Gupta, 2006, Musolino & Feehan, 2004; Reime et al., 2022). Associated with the underpinning theory, SLE activities and methods were founded on reciprocity, community engagement and cocreation. For example, Barker et al.’s (2022) service learning program had its foundations in the concept of cocreation as a collaborative and iterative process of designing, delivering and evaluating services in an equal and reciprocal relationship between service professionals, users and support networks. Based on pragmatism and participatory action research approaches, the process of cocreation was designed to connect the service with the Yolŋu Indigenous people’s ways of knowing, being and doing. Continuous reciprocal engagement occurred with valuing Yolŋu expertise and intellectual property; having diverse knowledge, practices, needs and aspirations; and honoring the health team’s workforce expertise in managing disabling consequences of disease and injury (Barker et al., 2022). However, this was described in relation to service learning principles rather than service leadership.

A few SLEs explicitly aligned theoretical and conceptual underpinnings with their learning and leadership structure. Mihaljevic et al. (2018) used a transparent methodology in their project description, with the intention of supporting understanding of the rationale behind their curriculum and replicability for others aiming to implement SLEs. The authors recognized that the SLE model alone (in this case, an IPTW) was not a clearly defined educational strategy. They therefore aimed to provide a concise and clear description of the context, underpinning rationale and frameworks to clarify their approach and implementation. The IPEC competency framework (IPEC, 2016), CanMEDS competencies (2015) and other frameworks were used in defining professional SLE activities and the organizational structure of their IPTW.

The explicitly reported leadership theory, model and frameworks in SLEs are summarized in Table 2 and further details in Expanded Data Table 2 in supplemental materials.

**Table 2:** Student leadership theory, model and frameworks

Author and year	Explicit student leadership theory, model or framework
Ahern and O’Donnell (2022)	Clinical operational, collaborative leadership
Beckman et al. (2022)	Canadian interprofessional health collaborative (CIHC) framework—collaborative leadership
Cox and Miranda (2003)	Linked leadership to systems theory and partnership with community
Pardo et al. (2022)	Shared leadership (similar to transformational leadership with mutual reciprocity) informed by principles of service learning
Donohue (2002)	Leadership styles, contingency theory of situational leadership style, intersection of leadership models and principles of coleadership
Foli et al. (2014)	Dimensions of leadership practice inventory, transformational leadership foundations, social change, service learning, civil responsibility
Goldstein et al. (2009)	Transformative theory of leadership (Bolman/Deals), servant leadership (Greenleaf)
Graber et al. (2017)	Leadership theories, Lencioni’s 5 dysfunctions of a team
Groh et al. (2011)	Servant leadership, social justice, civic responsibility
Gupta (2006)	Transformative leadership
Isaacson & Stacy (2004)	Management model—responsible for managing people to efficiently achieve organizational goals
Jack et al. 2022	Clinical leadership attributes: having interpersonal competence; possessing up to date clinical knowledge; being a positive role model
Leigh et al. (2019)	Coaching ideologies, promoting student nurse clinical leadership development and peer learning
Mihaljevic et al. (2018)	Alignment of CanMEDS, interprofessional competencies IPEC, Vygotsky’s proximal development zone and contact theory linked to leadership and learning
Morphet et al. (2014)	Leadership mentioned as part of competency standards for nursing, doctors
Reime et al. (2022)	Concept analysis of clinical leadership in nursing students highlighted interpersonal communication skills in contrast to task focused skills, which might be more readily linked with the development of management competence
Shields et al. (2013)	Self-regulated learning to guide student education leader

### Aiming for leadership: making the implicit explicit for learning objectives

As we reviewed the literature, an important element we discovered was the lack of explicit language that referred to leadership in learning aims and objectives. A learning objective is a description of what the learner must be able to do upon completion of an educational activity. A well-written learning objective outlines the knowledge, skills and/or attitudes that learners will gain from an educational activity and does so in a measurable way (Chatterjee & Corral, 2017). Learning objectives support instructional alignment so that learning objectives, assessment tools, and instructional methods mutually support the same educational outcome(s). Thus, the explicit integration of leadership in learning objectives can reveal the underlying conceptualization(s) of leadership in curriculum design. It can also reveal the prioritization of leadership as a primary or secondary goal of the learning activity.

Many papers did not report any learning objectives, with only nine of the 75 papers including or reporting explicit leadership objectives. Leadership appeared to be implicit rather than explicit in the objectives of student-led experiences, even when it was named in the terminology, definition or theory. It was at times a secondary objective but also may have been an implicit assumed component of other stated objectives such as professionalism, collaboration or community service. For example, Brewer and Stewart-Wynne (2013) included an objective that recognized the need for team interaction facilitation, conflict management and leadership as the fifth of six learning objectives for students in an IPTW. Others mentioned leadership as a broad aim or linked to specific SLE activities, e.g., developing leadership skills (Pardo et al., 2022; Graber et al., 2017).

In papers where leadership was more explicitly present as a primary focus, the SLE model was often a leadership skills academic-practice mixed course in which the SLE was a component. Goldstein et al. (2009) described a leadership course outline that integrated practice-based community projects. Two learning objectives were focused on leadership, with reference to underlying transformative leadership theory. These were “Understanding the importance of developing a transforming vision to guide leadership goals” and “Understanding the value of different leadership styles” (Goldstein et al., 2009, p. 755).

### Guiding leadership: the role of supervisors and facilitators

Our analysis of the data demonstrated a surprising lack of detail around guidance and support from supervisors and facilitators. Most papers either referred to or briefly described the supervision, mentorship or preceptor role, with 50 of 75 papers naming supervision but with a lack of clarity regarding the supervision model, approach or tasks. For example, students were able to contact preceptors to seek guidance (Foli et al., 2014), students had “appropriate support when needed while facilitating progressive independence” (Bostick et al., 2014, p. 514), faculty met weekly with students (Heath et al., 2019), or junior students were mentored by fourth-year students under the guidance of a faculty advisor (Rath et al. (2019)). In contrast to these less defined supervision approaches, Brewer & Stewart-Wynne (2013) described facilitating group learning and reflective sessions, encouraging end-of-day team debriefing, peer learning and supervisors taking a hands-off role when students were providing care services. Facilitators took an active role in students’ learning to encourage them to make decisions and implement actions themselves. Similarly, Nagelkerk et al. (2018) described staff preceptors conducting daily huddles, students reporting back to preceptors with potential diagnoses and proposing intervention plans, and preceptors supervising students’ phone calls with patients or providing guidance while students led patient education groups.

In some studies, students delivered an extension of care provided by staff with close supervision or joint planning and an active faculty presence. According to other studies, faculty members acted as consultants and were more distant from student activities, affording students a high level of autonomy. Kjaer et al. (2023) described the concept of autonomy-supportive teaching, based on the belief that intrinsic motivation can be stimulated through the facilitation of learner engagement, responsibility for one’s own learning, and the feeling of being competent in solving the task. They described autonomy-supportive supervision as developing student independence through supported responsibility, the building of rapport and personalized challenges. Specifically, they highlighted the importance of handing over decision-making through remote supervision so that students had to decide when to ask for support. Students were responsible for the consultations and would develop their awareness of their own limits. They also described the reciprocal process of explorative supervision, where clinical teachers take responsibility for direct/indirect guidance and where students take responsibility in patient encounters consistent with their capacity and commitment.

### Assessing and evaluating leadership

As we explored the descriptive nature of many SLE curricula, we were curious to see that there was an ambiguity around leadership assessment of students and evaluation of programs. Of the 75 papers reviewed, only 32 specifically mentioned assessment of students within the SLE, and only eight of these 32 papers mentioned the presence or nature of assessment related to leadership. Where described, assessment took a variety of forms. Performance-based assessments were reported in a small number of studies, either generally, for example, using the ‘usual placement assessment tool’ (O’Connor et al., 2019) or specifically, such as the Wisconsin Council on Occupational Therapy Education Evaluation of Student Performance for Level I Fieldwork (Neistadt & Cohn, 1990) or collaborative practice capabilities on the Interprofessional Capability Assessment (Brewer & Stewart-Wynne, 2013). Other assessments reported were summative feedback on projects (Graber et al., 2017), written assignments (Isaacson & Stacy, 2004), and perception/observational assessments (Mink et al., 2019). In some cases, assessment was reported as a formal measure of student learning in the curriculum, while in others, it was reported as a measure of research and program evaluation.

There were a few examples of direct student assessment of the presence or nature of leadership. These included self-assessments/reflections where students scrutinized their own leadership skills, roles, strengths, and methods (Reime et al., 2022). In SLEs that were part of a mixed academic/practice course, written assignments were used to allow them to document how they practiced leadership skills and to reflect further on leadership concepts taught in the course (Goldstein et al., 2009). In some SLEs, students completed a standardized leadership measure, for example, the Self-Assessment Leadership Instrument (Pardo, 2022). In one service learning SLE, students and preceptors were required to complete the Student Leadership Practices Inventory, which provides self-reports and observed assessments of behaviors across five leadership dimensions (Foli et al., 2014). For some IPTWs, assessments of IP competencies were completed using tools such as the Interprofessional Socialization and Valuing Scale and the Assessment for Interprofessional Team Collaboration Scale (Mink et al., 2021). However, these tools measure collaborative practice and teamwork, not collaborative leadership.

Though assessment was one part of curriculum integration, we reviewed data to explore if SLEs were explicitly part of or integrated into the requisite curriculum of the health professional program. In some cases, it was part of a mandatory requisite academic/practice mixed course, in others a separate community service project or a required elective.

We summarized curricular elements of SLEs in Table 2 along with other curricular elements of objectives, supervision, and assessment in the supplemental materials.

## Discussion

With the increase of SLEs in practice over the past decade, emerging formats and structures will be future templates for new SLEs and influence the development of students as current and future health care leaders. SLEs utilize and leverage student-led activities and experiences in health and social care with diverse forms of leadership. Thus, clinicians, educators and researchers would benefit from being attentive and intentional with respect to the evolution of SLEs and their design in the field. SLEs tend to have a common opportunistic origin, as they are often developed or accelerated in response to a system crisis (e.g., pandemic). This can be driven extrinsically from organizations or communities in need or intrinsically led by student initiative. For example: the Columbia COVID-19 Student Service had community response teams and student vaccination clinics initiatives that were primarily student initiated (Grilo et al., 2020). Whether organizationally or student driven, these types of initiatives can be nurtured and continually advanced to develop leadership, sustainable beyond an initial system catalyst.

Despite the expansion of SLE design and modeling in response to service needs, there does not appear to be equivalent attention given to the leadership development needs of the student. Through our review, we noted that many service learning definitions and concepts embrace the concept of a reciprocal model of service and learning in partnerships between communities and students (Petersen, 1994). Sigmon (1997) previously distinguished between service learning models in which the ‘service’ was prioritized and those in which the ‘learning’ was prioritized, suggesting that the needs of both the community and learners could be met in these models. Perhaps what is needed is a triad model where service, learning and leadership is prioritized. A key finding in our review was an apparent prioritization of attending to service delivery rather than the leadership development of students in SLEs. Whether the SLE was framed as a student-run clinic (O’Brien et al., 2015), an IPTW (Miheljevic et al., 2018) or a different model, there was often an absence of explicit leadership underpinning theory, concepts, objectives, facilitation or assessment. Although a minority of SLEs included more formalization and integration of leadership in their structures, leadership elements were implicit, hidden or absent in most others. This may not be surprising given the priority is clinical expertise development, where the linkages to leadership are not always recognized. There is an overlap of competency development in other learner development areas (collaboration, communication, advocacy, professionalism etc.). Perhaps this is an opportunity to not simply add on further leadership focus to a busy curriculum but to make explicit the leadership elements existing within. It may be both reasonable and feasible to leverage objectives that overlap in competency development and when not present, add explicit language and accountability regarding leadership development, not in isolation but in integration.

With respect to both service and leadership, SLEs can mutually develop leadership capacity among students and attend to gaps in health system capacity. Without this leadership development, SLEs are at risk of exploiting student services and labor without empowering their capacity for student leadership and learning. We note that these leadership aspects could simply have been underreported. Perhaps to support implementation in other settings, the focus of most papers was reporting more on the operational details of student-led activities than educational processes, such as curricular outlines, learning objectives or pedagogy, where leadership may have been more present. However, given that most SLEs originate as a response to service needs for communities and populations, it is critical that SLE administrators not lose sight of their intentions to also develop the leadership capacities of students providing that service. As recognized in the service learning literature, SLEs draw on a cost-effective, enthusiastic student workforce leading to mutual benefits for both students and organizations. However, we need to ensure that SLEs are structured and framed as a force for learning through a triad model of leadership, learning and service to develop future health professionals while simultaneously serving communities and health systems.

Another concept common across SLE roles and models was the enhanced autonomy that students had in SLEs compared with traditional practice activities. However, the level of autonomy varied widely from being supervised closely to being coached more remotely to limited supervision. As a whole, the reviewed papers commonly promoted the value of autonomy and independence in SLEs for students while simultaneously managing the level of risk through supervision. However, we noted that there was often a lack of detail about the presence and quality of facilitation and supervision related to leadership. In the absence of this detail, we were left with the impression that the SLE study authors believed that students should be left to learn through independence and less supervision. In response to this implicit assumption around leadership development, we propose that the level of autonomy and corresponding supervision should be tailored to align with the learning objectives, assessments and developmental level of students. Autonomy does not equal the absence of supervision, as students can be highly autonomous while having ready access to supervision and facilitation.

The descriptions of more explicit supervision practices in some SLEs, such as autonomy-supported and explorative supervision, could be seen as standard practice supervision and not dissimilar to supervision provided in other health professional student placements. This similarity itself is worth noting as it may be signaling a recognition that SLEs do not equal less supervision. Rather, SLEs can be supported by nuanced and responsive supervision that facilitates autonomy at a tailored rate through the provision of leadership opportunities and coaching. Responsive supervision in the SLE context may entail supervisors applying skilled judgment to scaffolding the extent, nature and amount of support offered. SLE supervisors can step back and forward where necessary, in a way that ensures autonomy support and champions student leadership practices. In some SLEs, rather than faculty acting as supervisors or managers, faculty members act as coaches or facilitators, being readily accessible yet promoting independence and autonomy (Kjaer et al., 2023; Nagelkerk et al., 2018). Nisbet et al. (2023), also describes a service-focussed placement model where students were encouraged to go beyond their comfort zone with the guidance and support of placement educators as needed. This model enhanced students’ competence to deliver services when supported by a higher level of trust from the supervisor but also resulted in high patient satisfaction, trust and confidence. This shifted the narrative from the students as a burden and potential risk to students as a benefit which provided higher quality safe care.

One area that was difficult to evaluate as both readers and reviewers of SLE literature was the level of curricular integration and the assessment context within which SLEs occur. Given our positionality as SLE leaders and managers, we feel that a focus on these aspects is relevant to educators and administrators seeking to apply and mobilize this knowledge of SLE design into their own settings. Given the importance of leadership development for students, it is important to determine the degree to which SLEs are integrated into the curriculum. With more detailed reporting of curricular elements, future studies of SLEs could support HPE faculty and administrators to integrate leadership development into their own contexts and curricula.

In their program description of their IPTW SLE, Mihaljevic et al. (2018) called for constructive alignment of learning goals and educational strategies with theory as a standard for these wards. If we want to ensure that leadership is a priority, it is important that this learning be driven by a similar standard, where learning concepts/definitions, theories, objectives, assessments and curricular integration guide SLE activities. In interpreting our data, we considered the emergence of five potential leadership student role profiles that were associated with SLE models and could be constructively aligned with theory and concepts. This was based on our examination and interpretation of the papers included, and though not a formal concept analysis, was based on mapping of explicit definitions, theory and concepts, informed by SLE experiences from our authorship team (Table 3).

**Table 3:** SLE student role profiles

Student role:	SLE definition	Concepts	SLE role example	Example references
Student as manager	Students being accountable for managing day-to-day operations of a clinic or service	Clinical leadership/managerial competence, interpersonal communication and patient safety/risk management	Student junior charge nurse in a unit or student administrator of a clinic	Ahern and O’Donnell (2022), Isaacson and Stacy, (2004), Jack et al., (2022), Leigh et al., (2019)
Student as facilitator	Students engaging in peer mentorship or facilitation/education in a health promotion patient group	Self-regulated learning, clinical foundations and experiential learning	Student peer mentor or group facilitator, health fairs or outreach group facilitation/education	Donohue (2002), Shields et al., (2013)
Student as collaborative leader	Students collaboratively working in a team and sharing decision making with other health professions in care	Interprofessional competencies, contact theory or social constructivism, coleadership and cocreation	Student team in a student-run clinic, interprofessional training ward	Beckman et al. (2022), Morphet et al., (2014)
Student as servant leader	Students providing a service to fill a system gap or support community needs	Service learning principles: servant leadership, community engagement, volunteerism, cultural competence	Service-learning projects/programs, community service	Goldstein et al., (2009), Groh et al., (2011)
Student as transformative leader	Students implementing a new service or initiative for transformation of individuals, environments, systems	Transformative leadership, cultural competence, social change and justice, critical reflection, cocreation	Advocacy, cocreation, quality improvement, transformative change	Pardo et al., (2022), Foli et al., (2014), Goldstein et al., (2009), Gupta (2006)

The definitions of leadership concepts proposed are linked to the roles that students take as leaders as opposed to the context or model in which the learning occurs (e.g., student-run clinic, IPTW, service learning course). With this interpretation of the concepts, leadership development is not just the service that the student provides but the type of leader the student strives to be. Multiple roles may be present and overlap in an operational model or context such as an SLE. For instance, a student can be a manager or collaborative and transformative leader in an SLE where he or she takes on multiple roles, e.g., a board member, a clinic manager, or a team member of a student-led clinic. This is in contrast to some student roles in SLEs, and where students implement or assist routine clinical services planned by supervisors or faculty, where we cannot identify a ‘student-led’ role outside of a normal student clinical role. Our hope is that these roles explicitly categorize the types of leadership and associated concepts, theories and activities that guide the process and outcomes of SLEs.

Our findings build on other literature reviews recognizing that student leadership definitions, theories and concepts are still underdeveloped and underexplored in HPE programs. Brewer et al. (2016) noted similar findings in IPE/practice programs, with only a quarter of the included papers reporting definitions, minimal use of leadership theory (9%) and, rarely, conceptual frameworks for interprofessional leadership (3%). Given the focus on interprofessional literature, Brewer and colleagues noted that the most common leadership approach was a collective (or collaborative) leadership model, with the second being transformational leadership. The authors noted the value of a student role as a competent collaborator, recognizing that student leadership activities in IPE can yield significant long-term benefits in the development of future student leaders in the workforce (Brewer & Stewart-Wynne, 2013; Hopkins, 2021). Nagel et al. (2022) noted a lack of common definitions and concepts for student-run health initiatives in the community. They also recognized that many concepts and terminology were used interchangeably with the root term “student-run”, such as “student-led” and “student-operated”. The authors distinguished among the various extents of students’ operational roles in SLEs and the involvement of academic institutions with 3 distinct conceptual definitions: student-run health initiatives, student-led health initiatives and student-infused health initiatives. These concepts were useful in determining the level of initiative, autonomy and partnership of students independently or in conjunction with academic institutions and curriculum. Although helpful for categorizing the role of students relative to faculty and institutional support, our study advances these conceptualizations by considering the role of student leadership within these various definitions. Finally, in reviews of student-led clinics, IPTWs and service learning, we noted that many of the SLEs’ terminology were rooted in the type of SLE structure used, such as a clinic, ward or community service. Our use of the term SLE is a first attempt at terminology that could include a wide spectrum of leadership activities based on leadership development and experiential learning rather than operational structures or settings.

Future directions for research in this field include the opportunity for conceptual analysis and realist evaluations to better understand the concepts and contexts of SLEs. A formal conceptual analysis may help determine and integrate leadership attributes and antecedents that could be categorized across different student roles to specific contexts beyond the scope of this review. Explicit leadership frameworks, such as LEADS, CanMEDS and CIHC leadership elements, could be used as an inductive interpretative tool in a concept analysis. These concepts can also be linked to broader research categories and paradigms such as cognitivist, behaviorist, and constructivist ways of knowing and teaching. Given the variety and complexity of SLE contexts and associated outcomes, a realist evaluation may be beneficial to future research to consider what SLE outcomes and processes work for which students, where/when and in what settings. To improve future understanding, more detail about the SLE context and author positionality in-text or supplemental material would support its application and replication in practice and further research analysis. As we have recommended through our review, curriculum integration, assessment, and objectives need to be explicit, and supplemental SLE activity/course outlines may also be helpful for understanding future research. We hope that this review will mobilize knowledge to develop criteria, guidelines and consensus statements to guide future SLE development internationally. To the authors’ knowledge, no such guidelines are currently available. From a practical standpoint, these overarching concepts can be further translated into guides and guidelines, specifically considering facilitation and faculty development for SLE implementation.

### Limitations

The limitations of the study include the difficulty in identifying and analyzing broad leadership concepts, the lack of SLE detail in reporting and the scope of the scoping review. The variation in leadership terminology and definitions made it difficult to create precise, all-inclusive search terms for databases, further complicated by how terms are indexed in databases. Typically, there is limited controlled vocabulary (e.g., Medical Subject Headings terms) available for topics that are new and emerging, less commonly or inconsistently searched. As a result, there was limited subject heading coverage available for the concept of SLEs and similar terms. With this limitation in mind and given the reliance on keyword searching, the capture of relevant SLE articles was dependent on how they were described, which, as we acknowledged in our paper, was variable and inconsistent. We also noted this in our initial literature search exploring SLE review papers. We attempted to account for these limitations by utilizing adjacency searching to address differences in possible phrasing. We did note that there were some articles that were not accessible for full-text review or may not have been reached in secondary searching. Given the representation of the literature we screened and the volume of data we collected, we feel this would likely have had little impact on our analysis and final outcomes. Though excluded for rationales mentioned earlier, we do recognize volunteer SLEs are seen as a way for advanced skill development, a valuable add on to mandatory curricula SLE opportunities and, in some centers, the only available SLE. Finally, potentially due to language limits, we also recognize the lack of studies in several parts of the world including Asia and South America as a limitation given the cultural aspects of leadership in the health professions and that leadership roles of students vary widely in these areas.

## Conclusion

This paper explores the concept of leadership in student-led experiences (SLEs). We used a scoping review methodology to better understand what is known about leadership concepts in the literature on SLEs and how these concepts are developed in practice-based curricula. A total of 75 studies met our scoping review study criteria and were included in the full-text review and extraction. Our findings advance other literature reviews, recognizing that student leadership definitions, theories and concepts are underdeveloped and underexplored in SLEs. This is of significance given the interest in and implementation of SLE models in education and practice. While responding to system gaps within health professional care, student-led experiences need to be supported by explicit leadership developmental theory/concepts/models and curricular pedagogy and assessment to support health professional education. In addition to leveraging a student workforce to address system needs, student-led experiences must also be a force for learning through a triad model of leadership, learning and service to develop future health professionals and leaders. There is an opportunity to add a leadership focus to existing curriculum with other aligned skills and competencies, not in isolation but in integration.

## Supplementary Information

Below is the link to the electronic supplementary material.

Supplementary file1 (DOCX 60 KB)

Supplementary file2 (DOCX 48 KB)
