# Supplementary material for: Exploring the “led” in health professional student-led experiences: a scoping review
Source: Adv Health Sci Educ Theory Pract. 2024 Oct 24;30(3):1007–36. doi: 10.1007/s10459-024-10355-x (PMC12119678; doi:10.1007/s10459-024-10355-x)
Supplement: Supplementary file 1 — Supplementary file1 (DOCX 60 KB) [file 10459_2024_10355_MOESM1_ESM.docx]

**Preferred Reporting Items for Systematic reviews and Meta-Analyses extension for Scoping Reviews (PRISMA-ScR) Checklist**

| **SECTION** | **ITEM** | **PRISMA-ScR CHECKLIST ITEM** | **REPORTED ON PAGE #** |
| --- | --- | --- | --- |
| **TITLE** | | | |
| Title | 1 | Identify the report as a scoping review. | 1 |
| **ABSTRACT** | | | |
| Structured summary | 2 | Provide a structured summary that includes (as applicable): background, objectives, eligibility criteria, sources of evidence, charting methods, results, and conclusions that relate to the review questions and objectives. | 2 |
| **INTRODUCTION** | | | |
| Rationale | 3 | Describe the rationale for the review in the context of what is already known. Explain why the review questions/objectives lend themselves to a scoping review approach. | 2-4 |
| Objectives | 4 | Provide an explicit statement of the questions and objectives being addressed with reference to their key elements (e.g., population or participants, concepts, and context) or other relevant key elements used to conceptualize the review questions and/or objectives. | 4 |
| **METHODS** | | | |
| Protocol and registration | 5 | Indicate whether a review protocol exists; state if and where it can be accessed (e.g., a Web address); and if available, provide registration information, including the registration number. | 4 |
| Eligibility criteria | 6 | Specify characteristics of the sources of evidence used as eligibility criteria (e.g., years considered, language, and publication status), and provide a rationale. | 5 |
| Information sources* | 7 | Describe all information sources in the search (e.g., databases with dates of coverage and contact with authors to identify additional sources), as well as the date the most recent search was executed. | 5 |
| Search | 8 | Present the full electronic search strategy for at least 1 database, including any limits used, such that it could be repeated. | 5 |
| Selection of sources of evidence† | 9 | State the process for selecting sources of evidence (i.e., screening and eligibility) included in the scoping review. | 5-6 |
| Data charting process‡ | 10 | Describe the methods of charting data from the included sources of evidence (e.g., calibrated forms or forms that have been tested by the team before their use, and whether data charting was done independently or in duplicate) and any processes for obtaining and confirming data from investigators. | 8 |
| Data items | 11 | List and define all variables for which data were sought and any assumptions and simplifications made. | 8 |
| Critical appraisal of individual sources of evidence§ | 12 | If done, provide a rationale for conducting a critical appraisal of included sources of evidence; describe the methods used and how this information was used in any data synthesis (if appropriate). | NA |
| Synthesis of results | 13 | Describe the methods of handling and summarizing the data that were charted. | 8 |
| **RESULTS** | | | |
| Selection of sources of evidence | 14 | Give numbers of sources of evidence screened, assessed for eligibility, and included in the review, with reasons for exclusions at each stage, ideally using a flow diagram. | 6 |
| Characteristics of sources of evidence | 15 | For each source of evidence, present characteristics for which data were charted and provide the citations. | 4-6 |
| Critical appraisal within sources of evidence | 16 | If done, present data on critical appraisal of included sources of evidence (see item 12). | NA |
| Results of individual sources of evidence | 17 | For each included source of evidence, present the relevant data that were charted that relate to the review questions and objectives. | 9-16 |
| Synthesis of results | 18 | Summarize and/or present the charting results as they relate to the review questions and objectives. | 9-16 |
| **DISCUSSION** | | | |
| Summary of evidence | 19 | Summarize the main results (including an overview of concepts, themes, and types of evidence available), link to the review questions and objectives, and consider the relevance to key groups. | 16-20 |
| Limitations | 20 | Discuss the limitations of the scoping review process. | 20 |
| Conclusions | 21 | Provide a general interpretation of the results with respect to the review questions and objectives, as well as potential implications and/or next steps. | 20 |
| **FUNDING** | | | |
| Funding | 22 | Describe sources of funding for the included sources of evidence, as well as sources of funding for the scoping review. Describe the role of the funders of the scoping review. | 20 |

JBI = Joanna Briggs Institute; PRISMA-ScR = Preferred Reporting Items for Systematic reviews and Meta-Analyses extension for Scoping Reviews.

* Where *sources of evidence* (see second footnote) are compiled from, such as bibliographic databases, social media platforms, and Web sites.

† A more inclusive/heterogeneous term used to account for the different types of evidence or data sources (e.g., quantitative and/or qualitative research, expert opinion, and policy documents) that may be eligible in a scoping review as opposed to only studies. This is not to be confused with *information sources* (see first footnote).

‡ The frameworks by Arksey and O’Malley (6) and Levac and colleagues (7) and the JBI guidance (4, 5) refer to the process of data extraction in a scoping review as data charting*.*

§ The process of systematically examining research evidence to assess its validity, results, and relevance before using it to inform a decision. This term is used for items 12 and 19 instead of "risk of bias" (which is more applicable to systematic reviews of interventions) to include and acknowledge the various sources of evidence that may be used in a scoping review (e.g., quantitative and/or qualitative research, expert opinion, and policy document).

*From:* Tricco AC, Lillie E, Zarin W, O'Brien KK, Colquhoun H, Levac D, et al. PRISMA Extension for Scoping Reviews (PRISMAScR): Checklist and Explanation. Ann Intern Med. 2018;169:467–473. [doi: 10.7326/M18-0850](http://annals.org/aim/fullarticle/2700389/prisma-extension-scoping-reviews-prisma-scr-checklist-explanation)

**Supplemental Table 1: Composition and Activities of SLEs**

| **Author and Year** | **Country** | **Study Type** | **health professional students** | **SLE Setting** | **SLE Activity** |
| --- | --- | --- | --- | --- | --- |
| Ahern, C., & O’Donnel, M. (2023) | International | synthesis | physiotherapy | clinics, community, hospital | physiotherapy clinical services and health promotion |
| Arlton, D. (1986) | United States | report | nursing, nutrition, communication disorders, human rehabilitative services, community health | ambulatory/outpatient | health promotion clinic |
| Barker, R., Witt, S., Bird, K., Stothers, K., Armstrong, E., Yunupingu, M. D., Marika, E. D., Brown, L., Moore, R., & Campbell, N. (2022) | Australia | mixed | occupational therapy, speech language pathology | community | allied health services to First Nations community |
| Beckman, E. M., Mandrusiak, A., Forbes, R., Mitchell, L., Tower, M., Cunningham, B., & Lewis, P. (2022) | Australia | mixed | counselling, exercise physiology, occupational therapy, physiotherapy and psychology | community | community based assessment and exercise program |
| Bennard, B., Wilson, J. L., Ferguson, K. P., & Sliger, C. (2004) | United States | report | medicine | community | rural community outreach clinic |
| Bostick, G., Hall, M., & Miciak, M. (2014) | Canada | qualitative | physiotherapy, kinesiology, pharmacy | ambulatory/outpatient | community rehabilitation clinic |
| Braun, B., Grünewald, M., Adam-Paffrath, R., Wesselborg, B., Wilm, S., Schendel, L., Hoenen, M., Müssig, K., & Rotthoff, T. (2019) | Germany | quantiative | medicine, nursing | hospital, university | Student providing nutritional management of patients in hospital unit |
| Brewer, M. L., & Stewart-Wynne, E. G. (2013) | Australia | quantiative | medicine, nursing, physiotherapy, occupational therapy, pharmacy, social work, dietetics, medical imaging, pharmacy | hospital | interprofessional inpatient ward duties, shared tasks such as medication, showering, wound dressing |
| Briggs, L., & Fronek, P. (2020) | International | synthesis | social work, physical therapy pharmacy, medical, other allied health disciplines | ambulatory/outpatient | clinical services, health promotion |
| Brown, D. A., & Ferrill, M. J. (2012) | United States | report | pharmacy | community | triaging patients, direct patient care - compounding and dispensing medications, and providing patient education for medical outreach mission |
| Childs, J. C., Sepples, S. B., & Moody, K. A. (2003) | United States | report | nursing | community, university | therapeutic mentoring/health promotion at-risk children |
| Clancy, G. P., & Duffy, F. D. (2013) | United States | perspective | medical, physician assistant, nursing, pharmacy, social work | primary care | annual interdisciplinary summer institute exploring firsthand social determinants of health, student-run PCMH clinics for the uninsured to teach systemsbased practice, team-based learning, and health system improvement |
| Cox, L. S., & Miranda, D. (2003) | United States | report | nursing | community | leadership community modules, community health activities, health promotion, education, health fairs |
| De Juan Pardo, M. Á., Vissandjee, B., Guillaumet Olives, M., Cerezuela Torre, M. Á., & Gallart Fernández-Puebla, A. (2022) | Spain | report | nursing | community, hospital | assessing health education needs of patient, family developing educational material |
| Donohue, M. V. (2002) | United States | perspective | occupational therapy | community, university | community-based program with psychosocial needs assessment and activities |
| Eudaley, S. T., Brooks, S. P., Jones, M. J., Franks, A. S., Dabbs, W. S., & Chamberlin, S. M. (2022) | United States | report | pharmacy | hospital | ensuring accurate medication reconciliation at transitions of care (TOC), medication optimization during hospitalization,generated a TOC discharge medication note |
| Foli, K. J., Braswell, M., Kirkpatrick, J., & Lim, E. (2014) | United States | quantiative | nursing | community, university | develop/implement public health fair with community, service learning grants writing/award |
| Forbes R. (2020) | Australia | qualitative | physiotherapy | ambulatory/outpatient | outpatient physiotherapy clinical services |
| Frakes, K, Brownie, S, Davies, L, Thomas, J, Miller M, Tyack, Z (2014) | Australia | report | exercise physiology, nutrition and dietetics, occupational therapy, pharmacy, podiatry, social work | ambulatory/outpatient | early intervention (consultations, intake screening clinics, case conferences) for clients with multiple chronic diseases |
| Freeth, D., Reeves, S., Goreham, C., Parker, P., Haynes, S., & Pearson, S. (2001) | United Kingdom | mixed | medicine, nursing, physical therapy, occupational therapy | hospital | interprofessional care, handover, reflection on training ward |
| Fröberg, M., Leanderson, C., Fläckman, B., Hedman-Lagerlöf, E., Björklund, K., Nilsson, G. H., & Stenfors, T. (2018) | Sweden | mixed | medicine, nursing, physiotherapy, occupational therapy, psychology | primary care | primary care team service |
| Goldstein, A. O., Calleson, D., Bearman, R., Steiner, B. D., Frasier, P. Y., & Slatt, L. (2009) | United States | report | medicine | community, university | service learning projects addressing community needs, networking, media, advocacy for underserved populations |
| Graber, K. C., Chodzko-Zajko, W., O’Connor, J. A., & Linker, J. M. (2017) | United States | synthesis | kinesiology | community, university | community-based wellness projects for the elderly |
| Grealish, L., Lucas, N., Neill, J., McQuellin, C., Bacon, R., & Trede, F. (2013) | Australia | mixed | nursing | long term care | resident care delivery, quality audit, family care planning meeting, leading medical rounds, wound and pain care management, induction activities for junior students |
| Grilo, S. A., Catallozzi, M., Desai, U., Sein, A. S., Quinteros Baumgart, C., Timmins, G., Edelman, D., & Amiel, J. M. (2020) | United States | perspective | medicine, public health, nurse practitioner, nutrition | community, hospital | service-learning projects developed, implemented supporting health system, community needs |
| Groh, C. J., Stallwood, L. G., & Daniels, J. J. (2011) | United States | quantiative | nursing | community, university | student led community projects |
| Gupta, J. (2006) | United States | report | occupational therapy, physical therapy, nursing, social work and education | community, university | family health services and education at shelter |
| Hamilton, V., Baird, K., & Fenwick, J. (2020) | Australia | qualitative | midwifery | ambulatory/outpatient | ante- and post natal care |
| Heath, J., Aker, R., Feld, H., Singer, R. L., & Norton, J. (2019) | United States | quantitative | dentistry, communication and information sciences, health sciences, nursing, pharmacy, public health, and social work | community | interprofessional oral health education |
| Hood, K., Cant, R., Leech, M., Baulch, J., & Gilbee, A. (2014) | Australia | mixed | nursing,medicine, physical therapy, occupational therapy | hospital | rehab ward care |
| Isaacson, J. J., & Stacy, A. S. (2004) | United States | perspective | nursing | hospital | expanded charge nurse role for junior students as staff, management liason, quality care, troubleshooting, decision-making |
| Jack, K., Bianchi, M., Costa, R. D. P., Grinberg, K., Harnett, G., Luiking, M.-L., Nilsson, S., & Scammell, J. M. E. (2022) | Scotland | synthesis | nursing | various | various in-situ clinical leadership nursing case examples |
| Jones, D., McAllister, L., Dyson, R., & Lyle, D. (2018)1 | Australia | qualitative | occupational therapy, speech language pathology | school | OT and SLP services targetting speech, language, communication, fine and gross motor skill delays |
| Jones, K., Blinkhorn, L. M., Schumann, S.-A., & Reddy, S. T. (2014) | United States | mixed | medicine | community | student-designed projects in response to community defined needs including referral mechanisms, educational tools, implementation of language specific screening tools for depression. |
| Kavannagh, J., Kearns, A., & McGarry, T. (2015) | Ireland | qualitative | occupational therapy, physical therapy, speech language pathology | primary care and range of community agencies | range of clinical rehabilitative services |
| Khan, T. M., & Jacob, S. A. (2015) | Malaysia | report | pharmacy | community | community engagement and service activities |
| Kjær, L. B., Nielsen, K.-J. S., Christensen, M. K., & Strand, P. (2023) | Denmark | mixed | medicine and interprofessional | community, hospital | outpatient student clinic, student ward |
| Lachmann, H., Ponzer, S., Johansson, U.-B., Benson, L., & Karlgren, K. (2013) | Sweden | mixed | medicine, nursing, physical therapy, occupational therapy | hospital | Wound care, admin meds, mobilization, blood pressure monitoring, planning patient care |
| Leigh, J., Littlewood, L., & Lyons, G. (2019) | United Kingdom | perspective | nursing | hospital | patient care, shift handover - coaching approach for clinical leadership |
| Lestari, E., Scherpbier, A., & Stalmeijer, R. (2020) | Indonesia | mixed | medicine, midwifery, nursing | community | Students conduct a community health-problem survey, analysing the data to diagnose primary community health problems and determining and implementing interventions for the respective problems. |
| Longman, J. M., Barraclough, F., & Swain, L. S. (2020) | Australia | report | occupational therapy, physical therapy, speech language pathology | community rural | addressing gaps with care services in rural schools and aged care facilities, cultural awareness training, quality improvement projects |
| McGettigan, P., & McKendree, J. (2015) | United Kingdom | mixed | medicine, nursing, occupational therapy, physical therapy | hospital | students responsible for 4–5 patients caseload, all aspects of care, assisting with therapy sessions, going on home visits, undertaking tests and clinical observations, participating in medication rounds and ward rounds. |
| Mihaljevic, A. L., Schmidt, J., Mitzkat, A., Probst, P., Kenngott, T., Mink, J., Fink, C. A., Ballhausen, A., Chen, J., Cetin, A., Murrmann, L., Müller, G., Mahler, C., Götsch, B., & Trierweiler-Hauke, B. (2018) | Germany | report | medicine, nursing, interprofessional health students | hospital | work together in interprofessional (IP) teams and manage the full responsibility for the medical treatment and rehabilitation of real life patients. |
| Mink, J., Mitzkat, A., Krug, K., Mihaljevic, A., Trierweiler-Hauke, B., Götsch, B., Wensing, M., & Mahler, C. (2021) | Germany | mixed | medicine, nursing | hospital | patient care tasks of 6 patients in 2 hosptial rooms |
| Morphet, J., Hood, K., Cant, R., Baulch, J., Gilbee, A., & Sandry, K. (2014) | Australia | mixed | nursing, medicine and other health care students | hospital | assess patients, plan, manage care, investigations, referral, and discharge in emergency and rehabilitation ward |
| Musolino, G. M., & Feehan, P. (2004) | United States | report | physical therapy | community | community engagement to increase awareness of physical therapy, promoting active participation within a mentor/protégé framework |
| Nagelkerk, J., Thompson, M. E., Bouthillier, M., Tompkins, A., Baer, L. J., Trytko, J., Booth, A., Stevens, A., & Groeneveld, K. (2018) | United States | mixed | medicine, pharmacy, physician assistant | primary care clinic | interprofessional collaborative practice, daily huddles, focus patient visits, phone calls, team-based case presentations, medication reconciliation, and student-led group diabetes education classes. |
| Neistadt, M. E., & Cohn, E. S. (1990) | United States | report | occupational therapy | hospital, community | student-led independent living skills groups |
| Ng, E., & Hu, T. (2017) | Canada | report | various | ambulatory/outpatient | student-run free clinics providing interprofessional health care to underserved populations |
| Nicole, M., Fairbrother, M., Nagarajan, S.V., Blackford, J., Sheepway, L., Penman, M., & McAllister, L. (2015) | Australia | report | physical therapy | hospital | students manage ward. prioritizing patient lists and new patient referrals, developing and running a daily exercise program, arranging assistance from therapy assistants,communicating with nursing, medical, team |
| O’Brien, D., Swann, J., & Heap, N. (2015) | New Zealand | perspective | counselling psychology, nursing, occupational therapy, oral health, physical therapy, podiatry, psychotherapy, speech and language therapy | community, university | student-led clinic collaborative person-centred healthcare services |
| O’Connor, A., Liston, E., & O’Donnell, M. (2019) | Ireland | qualitative | physical therapy | community | evidence-based health promotion programmes to community groups |
| Oosterom, N., Floren, L. C., Ten Cate, O., & Westerveld, H. E. (2019) | International | synthesis | various | hospital | interprofessional training ward services |
| Palombaro, K. M., Dole, R. L., & Lattanzi, J. B. (2011) | United States | report | physical therapy | ambulatory/outpatient | physical therapy services for members without insurance or benefits, a center for health and wellness education and screening for community residents. |
| Paparella-Pitzel, S., Anderson, E. Z., Rothpletz-Puglia, P., & Parrott, J. S. (2021) | United States | mixed | physical therapy | ambulatory/outpatient | rehab services, assessment, examination, peer teaching |
| Patterson, F., Fleming, J., Marshall, K., & Ninness, N. (2017) | Australia | qualitative | occupational therapy | hospital | student led group programs extending therapy - meal preparation, community access, upper limb, cognitive |
| Peterson, S. J., & Schaffer, M. J. (1999) | United States | perspective | nursing | community | public health projects to meet service needs of community agencies |
| Powers, C. A., Thomson, C. C., Feuerstein, I., Cross, M., Powers, E. M., Prout, M., & Geller, A. C. (2008) | United States | report | medicine | community | student led smoking prevention programs for school students |
| Rath, C., Tillman, F., Stickel, J., Jones, M., & Armistead, L. (2019). Implementation of a Student-Developed, Service-Based Internship for Pharmacy Students. Innovations in Pharmacy, 10(2), 10.24926/iip.v10i2.1550. https://doi.org/10.24926/iip.v10i2.1550 | United States | report | pharmacy | community | ambulatory care of underserved populations; optimal medication therapy, counselling and telephone follow up on prescriptions, point of care screening and facilitated referrals, quality improvement projects |
| Reeves, S., Freeth, D., McCrorie, P., & Perry, D. (2002) | United Kingdom | mixed | medicine, nursing, occupational therapy, physical therapy | hospital | students plan and deliver quality interprofessional care in training ward |
| Reime, M. H., Bell, K., Albrigtsen, B., & Beisland, E. G. (2022) | Norway | qualitative | nursing | hospital | care of surgical and medical patients |
| Richards, E. A., Novak, J. C., & Davis, L. V. (2009) | United States | report | nursing | community | disaster relief, family health clinic |
| Rupert, D. D., Alvarez, G. V., Burdge, E. J., Nahvi, R. J., Schell, S. M., & Faustino, F. L. (2022) | United States | perspective | medicine | ambulatory/outpatient | student clinic roles: as medical care providers, as medical education,as advocacy organizations. |
| Schneider, A. R., Stephens, L. A. M., Ochoa Marín, S. C., & Semenic, S. (2018) | Canada | qualitative | nursing | community | community diagnostics, home visits, and health education sessions |
| Shields, N., Bruder, A., Taylor, N. F., & Angelo, T. (2013) | Australia | qualitative | physical therapy | community | student-led progressive resistance program |
| Simmons, K., Klein, M., Stevens, C., & Jacobson, T. (2019) | United States | report | pharmacy | primary care | diabetic management, scheduling/leading clinic visits,documentation and follow up |
| Smith, B. D., Marshall, I., Anderson, B. E., & Daniels, K. K. (2017) | United States | report | social work | community | community appreciation day, international service learning project |
| …………………….. | Australia | report | nursing | community | day-to-day clinic operation, advertisement ,marketing, student orientation materials, triage, assessment, testing, diagnosis, treatment, prescriptions, education and coaching. |
| Tokolahi, E., Broman, P., Longhurst, G., Pearce, A., Cook, C., Andersen, P., & Brownie, S. (2021) | New Zealand | synthesis | various | community | student-led health services |
| Tsang, E. S., Cheung, C. C., & Sakakibara, T. (2016) | Canada | quantative | medicine, nursing, pharmacy, physical therapy, and dietetics. | community | designed educational materials, attended team-building exercises and conductded outreach sessions |
| Tyndall, D. E., Kosko, D. A., Forbis, K. M., & Sullivan, W. B. (2020) | United States | report | nursing | community | student-led lectures, teaching of primary school children about service learning who conducted service projects aimed to collect supplies |
| VanGraafeiland, B., Sloand, E., Silbert-Flagg, J., Gleason, K., & Dennison Himmelfarb, C. (2019) | United States | report | nursing | hospital | quality improvement projects |
| Vijn, T., Fluit, C., Kremer, J., Faber, M., & Wollersheim, H. (2017) | International | synthesis | medicine | various | student-led patient education, service delivery |
| Voss, H. C. (2016) | United States | qualitative | nursing | community | community based service learning project |
| Wilson, O. W., Broman, P., Tokolahi, E., Andersen, P., & Brownie, S. (2023) | International | synthesis | various | ambulatory/outpatient | student clinic services |

**Expanded Data Table 2: SLE Terminology, Definition, Theory, Concept, Framework**

| **Author and Year** | **SLE Terminology** | **SLE definition (N=No definition of terminology present)** | **Leadership development described in definition (Y=Yes, N=No)** | **Explicit student leadership theory, concept or framework** | **Leadership Theory/Concept/Framework Present** |
| --- | --- | --- | --- | --- | --- |
| Ahern, C., & O’Donnel, M. (2023) | student-led services | student-led services involves students being responsible for the day-to-day running of the service or clinic along with caseload management. | N | clinical operational, collaborative leadership | Y |
| Arlton, D. (1986) | health promotion clinic | N | N | N | N |
| Barker, R., Witt, S., Bird, K., Stothers, K., Armstrong, E., Yunupingu, M. D., Marika, E. D., Brown, L., Moore, R., & Campbell, N. (2022) | student-implemented service | student-implemented allied health services where students on clinical placement take primary responsibility  for delivery of services,17 | N | pragmatic co-creation process, derived from participatory action research approaches, continuous reciprocal engagement to Yolŋu ways of knowing, being and doing | N |
| Beckman, E. M., Mandrusiak, A., Forbes, R., Mitchell, L., Tower, M., Cunningham, B., & Lewis, P. (2022) | interprofessional student-led clinic | interprofessional student-led clinics have been established to build capacity beyond traditional single-discipline placements, facilitate development of collaborative approaches to healthcare and address community healthcare needs | N | canadian interprofessional health collaborative (CIHC) framework - collaborative leadership | Y |
| Bennard, B., Wilson, J. L., Ferguson, K. P., & Sliger, C. (2004) | student-run rural outreach clinic | student-conducted experiences rural outreach clinic | N | N | N |
| Bostick, G., Hall, M., & Miciak, M. (2014) | student led clinics | student led clinics are defined as clinical learning centres run by students to enhance the heatlh of a community | N | N | N |
| Braun, B., Grünewald, M., Adam-Paffrath, R., Wesselborg, B., Wilm, S., Schendel, L., Hoenen, M., Müssig, K., & Rotthoff, T. (2019) | IP Teaching and Learning Unit | N | N | interprofessional education, allports content hypothesis, inquiry/research oriented learning | N |
| Brewer, M. L., & Stewart-Wynne, E. G. (2013) | IP Training Ward | interprofessional groups of students undertaking a 2-3 week placement developing interprofessional practice capabilities | N | interprofessional capability framework | N |
| Briggs, L., & Fronek, P. (2020) | Student-led health clinic | student-led clinics defined as those health clinics where services are delivered by students under the supervision of qualified educators who are appropriately licensed or accredited | Y | N | N |
| Brown, D. A., & Ferrill, M. J. (2012) | Advanced Pharmacy Practice Experience (APPE) Rotation | N | N | N |  |
| Childs, J. C., Sepples, S. B., & Moody, K. A. (2003) | Service learning course | youth mentoring program as a service learning course | N | N | N |
| Clancy, G. P., & Duffy, F. D. (2013) | student-led free clinics | N |  | patient care medical home model | N |
| Cox, L. S., & Miranda, D. (2003) | community leadership modules | leadership modules linked to system theory | Y | linked leadership to systems theory and partnership with community | Y |
| De Juan Pardo, M. Á., Vissandjee, B., Guillaumet Olives, M., Cerezuela Torre, M. Á., & Gallart Fernández-Puebla, A. (2022) | student-led dedicated education unit | dedicated education units - academic partnership between a clinical site and the educational institution, optimizing clinical learning environment making health education accessible to a changing and vulnerable population | N | shared leadership (similar to transformational leadership with mutual reciprocity) informed by principles of service learning | Y |
| Donohue, M. V. (2002) | group co-leadership | group co-leadership roles in community based program | Y | leadership styles, contingency theory of situational leadership style, intersection of leadership models and principles of co-leadership | Y |
| Eudaley, S. T., Brooks, S. P., Jones, M. J., Franks, A. S., Dabbs, W. S., & Chamberlin, S. M. (2022) | student-driven transitions of care | N | N | N | N |
| Foli, K. J., Braswell, M., Kirkpatrick, J., & Lim, E. (2014). Development of Leadership Behaviors in Undergraduate Nursing Students: A Service-Learning Approach. Nursing Education Perspectives, 35(2), 76. https://doi.org/10.5480/11-578.1 | service learning course | leadership management and service learning course | Y | dimensions of leadership practice inventory, transformational leadership foundations, social change,service learning, civil responsibiity | Y |
| Forbes R. (2020) | student-led clinics | student-led clinic refers to clinical learning centers where students manage and deliver supervised health care as well as contribute to the operational management | N | N |  |
| Frakes, K, Brownie, S, Davies, L, Thomas, J, Miller M, Tyack, Z (2014) | student-assisted clinical service | N | N | N | N |
| Freeth, D., Reeves, S., Goreham, C., Parker, P., Haynes, S., & Pearson, S. (2001) | interprofessional training ward | N | N | N | N |
| Fröberg, M., Leanderson, C., Fläckman, B., Hedman-Lagerlöf, E., Björklund, K., Nilsson, G. H., & Stenfors, T. (2018) | student-run clinic | student-run clinic -active learning opportunities in authentic settings, student acts as the main provider of care, with support from the clinical supervisor | N | N | N |
| Goldstein, A. O., Calleson, D., Bearman, R., Steiner, B. D., Frasier, P. Y., & Slatt, L. (2009) | leadership skills based course | advanced leadership skills in community service course - model for training undergraduate medical students in leadership skills. | Y | transformative theory of leadership (Bolman/Deals), servant leadership (Greenleaf) | Y |
| Graber, K. C., Chodzko-Zajko, W., O’Connor, J. A., & Linker, J. M. (2017) | service learning | service learning involvement of students in community while engaged in academic learning | N | leadership theories, Lencioni's 5 dysfunctions of a team | Y |
| Grealish, L., Lucas, N., Neill, J., McQuellin, C., Bacon, R., & Trede, F. (2013) | student nurse led ward | student nurse led ward - model of clinical education with student-led residential aged care and enhance student learning through a ‘communities of practice’ learning architecture | N | community of practice | N |
| Grilo, S. A., Catallozzi, M., Desai, U., Sein, A. S., Quinteros Baumgart, C., Timmins, G., Edelman, D., & Amiel, J. M. (2020) | service learning organization | N | N | preparation, action, reflection, and evaluation (PARE) service learning model | N |
| Groh, C. J., Stallwood, L. G., & Daniels, J. J. (2011) | service learning | service learning linking students and faculty with area development organizations. | N | servant leadership,social justice, civic responsibility | Y |
| Gupta, J. (2006) | service learning definition | service learning - CCHPS definition - client-centeredness, reciprocity, reflective practice, social responsibility | N | transformative learning, critical reflection, service learning, interdisciplinary collaboration, cultural competence | Y |
| Hamilton, V., Baird, K., & Fenwick, J. (2020) | student led antenatal clinic | N | N | N | N |
| Heath, J., Aker, R., Feld, H., Singer, R. L., & Norton, J. (2019) | interprofessional service learning program | N | N | interprofessional education collaborative (IPEC) competencies | N |
| Hood, K., Cant, R., Leech, M., Baulch, J., & Gilbee, A. (2014) | student-led hospital ward | N | N | N | N |
| Isaacson, J. J., & Stacy, A. S. (2004) | Expanded charge nurse | management course mimicing management and leadership duties that a charge nurse would have for staff nurses | Y | management model - responsible for managing people to efficiently achieve organizational goals (Marquis and Huston management process function - planning, organizing, staffing, directing , controlling | Y |
| Jack, K., Bianchi, M., Costa, R. D. P., Grinberg, K., Harnett, G., Luiking, M.-L., Nilsson, S., & Scammell, J. M. E. (2022) | clinical leadership | N | N | clinical leadership attributes: having interpersonal competence;possessing up to date clinical knowledge; being a positive role model | Y |
| Jones, D., McAllister, L., Dyson, R., & Lyle, D. (2018)1 | student-led services | service learning partnerships | N | N | N |
| Jones, K., Blinkhorn, L. M., Schumann, S.-A., & Reddy, S. T. (2014) | service learning elective, student-run free clinics | mutually beneficial balance of community service and student learning | N | SERVE (Service, Education, Reflection, Volunteerism Elective) model | N |
| Kavannagh, J., Kearns, A., & McGarry, T. (2015) | student-led clinics | Student-led clinics are a mode of healthcare delivery in which medical, nursing and or healthcare professional students take primary responsibility for the ‘logistics & operational management’ of the clinic | Y | N | N |
| Khan, T. M., & Jacob, S. A. (2015) | service learning in elective community engagement unit | service learning defined as teaching and learning strategy that incorporates meaningful community service with instruction and reflection to enrich the learning experience, teach civic responsibility, and strengthen communities. | N | service learning | N |
| Kjær, L. B., Nielsen, K.-J. S., Christensen, M. K., & Strand, P. (2023) | student clinic, ward | Student clinic enable authentic student-patient learning relations through enhanced student responsibility. | N | patient-centred learning, person-centred and autonomy-supported practices, professionialism | N |
| Lachmann, H., Ponzer, S., Johansson, U.-B., Benson, L., & Karlgren, K. (2013) | interprofessional training ward | IPE defined but not IPTW | Y | shared understanding of interprofessional teamwork: roles and responsibilities, communication, learning and reflection, the patient/client and ethics and attitudes. | N |
| Leigh, J., Littlewood, L., & Lyons, G. (2019) | Student nurse clinical leadership | N | N | GM Synergy based upon coaching ideologies, placing emphasis on delivering patient-centred care, promoting student nurse clinical leadership development and peer learning | Y |
| Lestari, E., Scherpbier, A., & Stalmeijer, R. (2020) | Community based interprofessional education | N | N | N |  |
| Longman, J. M., Barraclough, F., & Swain, L. S. (2020) | community based work ready placements, service learning | N | N | N | N |
| McGettigan, P., & McKendree, J. (2015) | interprofessional training placement | ‘Linkoping model’ interprofessional training ward | N | N | N |
| Mihaljevic, A. L., Schmidt, J., Mitzkat, A., Probst, P., Kenngott, T., Mink, J., Fink, C. A., Ballhausen, A., Chen, J., Cetin, A., Murrmann, L., Müller, G., Mahler, C., Götsch, B., & Trierweiler-Hauke, B. (2018) | Interprofessional training ward | IPTW are an interprofessional educational intervention which aim to enable students and trainees from different health professions to work self-responsibly in order to manage the medical treatment and rehabilitation of real-life patients together as an interprofessional team. | Y | theoretical background IP competency frameworks/principles, adult learning, Vygotsky's zone of proximal development, Complexity and Contact theory | Y |
| Mink, J., Mitzkat, A., Krug, K., Mihaljevic, A., Trierweiler-Hauke, B., Götsch, B., Wensing, M., & Mahler, C. (2021) | IP training ward | IPTW enable practical training in complex health care settings and facilitate the development of interprofessional competencies within interprofessional collaboration experiences | Y | competency-based interprofessional education, identity | N |
| Morphet, J., Hood, K., Cant, R., Baulch, J., Gilbee, A., & Sandry, K. (2014) | IP training ward | training ward in which preregistration health care students work in inter professional teams, while under supervision, to manage the care of patients | N | leadership part of IP competency standards for nursing, doctors | Y |
| Musolino, G. M., & Feehan, P. (2004) | service learning | Service learning defined the accomplishment of tasks that meet genuine human needs in combination with conscious educational growth | N | mentoring (coaching) and pluralism (cultural competence) theoretical constructs, service learning: Kolb’s Experiential Learning , Dewey Democracy of Inquiry, Schön Reflective Practice. Boyers model of faculty community engagement | N |
| Nagelkerk, J., Thompson, M. E., Bouthillier, M., Tompkins, A., Baer, L. J., Trytko, J., Booth, A., Stevens, A., & Groeneveld, K. (2018) | interprofessional collaborative practice program | interprofessional collaborative practice | N | identified paradim of interprofessional education and practice | N |
| Neistadt, M. E., & Cohn, E. S. (1990) | student-led groups | N | N | N | N |
| Ng, E., & Hu, T. (2017) | student run free clinic | student-run free clinics defined as organizations composed of students from a variety of disciplines [professions] that collaboratively plan and delivervhealthcare and health promotion services with the supervisionvand assistance of licensed healthcare professionals | Y | N | N |
| Nicole, M., Fairbrother, M., Nagarajan, S.V., Blackford, J., Sheepway, L., Penman, M., & McAllister, L. (2015) | student-led services | N | Y | N | N |
| O’Brien, D., Swann, J., & Heap, N. (2015) | interprofessional student-led health clinic | N | N | Wenger community of practice | N |
| O’Connor, A., Liston, E., & O’Donnell, M. (2019) | student-led groups | student-led placement | N | N | N |
| Oosterom, N., Floren, L. C., Ten Cate, O., & Westerveld, H. E. (2019) | interprofessional training ward | interprofessional training ward defined as an in-patient clinical ward where students from more than one health care professionare collaboratively responsible for patient care. | N | N |  |
| Palombaro, K. M., Dole, R. L., & Lattanzi, J. B. (2011) | student-led pro-bono clinic | N | N | leadership development program but no details of theory/model/framework |  |
| Paparella-Pitzel, S., Anderson, E. Z., Rothpletz-Puglia, P., & Parrott, J. S. (2021) | student-run pro bono clinic | N | N | peer-assisted learning | N |
| Patterson, F., Fleming, J., Marshall, K., & Ninness, N. (2017) | student-led service | student-led service approach described as model for professional practice education | N | peer learning; student-led service approach | N |
| Peterson, S. J., & Schaffer, M. J. (1999) | service learning | service learning is a reciprocal relationship between students and communities in which both parties engage in service and learning. | N | group collaboration, reflection, reciprocity | N |
| Powers, C. A., Thomson, C. C., Feuerstein, I., Cross, M., Powers, E. M., Prout, M., & Geller, A. C. (2008) | service learning | None | N | tobacco control competencies | N |
| Rath, C., Tillman, F., Stickel, J., Jones, M., & Armistead, L. (2019). Implementation of a Student-Developed, Service-Based Internship for Pharmacy Students. Innovations in Pharmacy, 10(2), 10.24926/iip.v10i2.1550. https://doi.org/10.24926/iip.v10i2.1550 | student-directed practicum | not stated | N | service learning | N |
| Reeves, S., Freeth, D., McCrorie, P., & Perry, D. (2002) | interprofessional training ward | training ward origin | N | interprofessional learning | N |
| Reime, M. H., Bell, K., Albrigtsen, B., & Beisland, E. G. (2022) | student-run teams | Student-run teams organize clinical studies to ease the transition from the student role to the professional nursing role | N | concept analysis of clinical leadership in nursing students  highlighted interpersonal communication skills in contrast  to task focused skills, which might be more readily linked  with the development of management competence | Y |
| Richards, E. A., Novak, J. C., & Davis, L. V. (2009) | service learning | service learning is a structured reciprocal learning experience | N | N | N |
| Rupert, D. D., Alvarez, G. V., Burdge, E. J., Nahvi, R. J., Schell, S. M., & Faustino, F. L. (2022) | student run free clinics | desribe mission of student-run free clinics to provide health care to uninsured individuals and to educate and empower the next generation of medical trainees to care via service-learning mechanism | N | N | N |
| Schneider, A. R., Stephens, L. A. M., Ochoa Marín, S. C., & Semenic, S. (2018) | service learning | service-learning is an innovative model of nursing education and community development that aims to engage students with communities in ways that enhance their academic experiences while simultaneously serving the needs of the community | N | service learning pedagogy | N |
| Shields, N., Bruder, A., Taylor, N. F., & Angelo, T. (2013) | student-led exercise program | program description: students led the exercise program | N | self-regulated learning model | Y |
| Simmons, K., Klein, M., Stevens, C., & Jacobson, T. (2019) | student pharmacist-run diabetes education/management clinic | N | N | N | N |
| Smith, B. D., Marshall, I., Anderson, B. E., & Daniels, K. K. (2017) | service learning | service learning defined as pedagogical approach that integrates students’ classroom instruction with community experience | N | Deweyism theory for service learning, PREPARE model for macro-social work practice with communities and organization | N |
| Stuhlmiller, C. M., & Tolchard, B. (2015) | student led clinic | N | N | N | N |
| Tokolahi, E., Broman, P., Longhurst, G., Pearce, A., Cook, C., Andersen, P., & Brownie, S. (2021) | student-led clinics | Student leadership not named but student did lead programs | N | operational underpinning models of practice - business/private, project-based, educational model - community of practice, interprofessional care, client centred | N |
| Tsang, E. S., Cheung, C. C., & Sakakibara, T. (2016) | student-led community service | N | N | none stated | N |
| Tyndall, D. E., Kosko, D. A., Forbis, K. M., & Sullivan, W. B. (2020) | service learning | Service-learning has been identified as a component of community and acdemic partnerships, structured learning experiences that combine community service with contextual learning to foster citizenship. | N | service learning model referenced | N |
| VanGraafeiland, B., Sloand, E., Silbert-Flagg, J., Gleason, K., & Dennison Himmelfarb, C. (2019) | service based learning program | not stated | N | principles of QI and safety | N |
| Vijn, T., Fluit, C., Kremer, J., Faber, M., & Wollersheim, H. (2017) | student-provided, student run patient education clinics | na | N | N | N |
| Voss, H. C. (2016) | service learning | Service-learning is a teaching–learning strategy in higher education that provides hands-on experiences in authentic clinical environments. | N | grounded in service learning principles | N |
| Wilson, O. W., Broman, P., Tokolahi, E., Andersen, P., & Brownie, S. (2023) | student run clinic | student-run clinics offer students the opportunity to gain early clinical and leadership experience via direct engagement in healthcare delivery, may be linked with service-learning | Y | N | N |

**Supplemental Table 2: Curricular Elements of SLE**

**Legend (Y=Yes, N=No)**

| **Author and Year** | **Supervision Model Described** | **Leadership Objectives Described** | **Assessment Described** | **Leadership Assessment Described** | **Curriculum Integration Described** |
| --- | --- | --- | --- | --- | --- |
| Ahern, C., & O’Donnel, M. (2023) | Y | N | N | N | N |
| Arlton, D. (1986) | N | N | N | N | Y |
| Barker, R., Witt, S., Bird, K., Stothers, K., Armstrong, E., Yunupingu, M. D., Marika, E. D., Brown, L., Moore, R., & Campbell, N. (2022) | Y | N | Y | N | Y |
| Beckman, E. M., Mandrusiak, A., Forbes, R., Mitchell, L., Tower, M., Cunningham, B., & Lewis, P. (2022) | Y | N | N | N | Y |
| Bennard, B., Wilson, J. L., Ferguson, K. P., & Sliger, C. (2004) | Y | N | N | N | Y |
| Bostick, G., Hall, M., & Miciak, M. (2014) | Y | N | N | N | Y |
| Braun, B., Grünewald, M., Adam-Paffrath, R., Wesselborg, B., Wilm, S., Schendel, L., Hoenen, M., Müssig, K., & Rotthoff, T. (2019) | N | N | N | N | Y |
| Brewer, M. L., & Stewart-Wynne, E. G. (2013) | Y | Y | Y | N | Y |
| Briggs, L., & Fronek, P. (2020) | N | N | N | N | N |
| Brown, D. A., & Ferrill, M. J. (2012) | N | N | Y | N | Y |
| Childs, J. C., Sepples, S. B., & Moody, K. A. (2003) | N | N | Y | N | Y |
| Clancy, G. P., & Duffy, F. D. (2013) | N | N | N | N | N |
| Cox, L. S., & Miranda, D. (2003) | Y | N | Y | Y | Y |
| De Juan Pardo, M. Á., Vissandjee, B., Guillaumet Olives, M., Cerezuela Torre, M. Á., & Gallart Fernández-Puebla, A. (2022) | Y | Y | Y | Y | Y |
| Donohue, M. V. (2002) | N | Y | Y | N | Y |
| Eudaley, S. T., Brooks, S. P., Jones, M. J., Franks, A. S., Dabbs, W. S., & Chamberlin, S. M. (2022) | Y | N | Y | N | Y |
| Foli, K. J., Braswell, M., Kirkpatrick, J., & Lim, E. (2014)\ | Y | Y | N | N | Y |
| Forbes R. (2020) | Y | N | N | N | y |
| Frakes, K, Brownie, S, Davies, L, Thomas, J, Miller M, Tyack, Z (2014) | Y | N | N | N | Y |
| Freeth, D., Reeves, S., Goreham, C., Parker, P., Haynes, S., & Pearson, S. (2001) | Y | N | N | N | N |
| Fröberg, M., Leanderson, C., Fläckman, B., Hedman-Lagerlöf, E., Björklund, K., Nilsson, G. H., & Stenfors, T. (2018) | Y | Y | N | N | Y |
| Goldstein, A. O., Calleson, D., Bearman, R., Steiner, B. D., Frasier, P. Y., & Slatt, L. (2009) | N | Y | Y | Y | Y |
| Graber, K. C., Chodzko-Zajko, W., O’Connor, J. A., & Linker, J. M. (2017) | Y | Y | Y | Y | Y |
| Grealish, L., Lucas, N., Neill, J., McQuellin, C., Bacon, R., & Trede, F. (2013) | Y | N | Y | N | Y |
| Grilo, S. A., Catallozzi, M., Desai, U., Sein, A. S., Quinteros Baumgart, C., Timmins, G., Edelman, D., & Amiel, J. M. (2020) | Y | N | N | N | Y |
| Groh, C. J., Stallwood, L. G., & Daniels, J. J. (2011) | N | N | Y | Y | Y |
| Gupta, J. (2006) | Y | N | N | N | Y |
| Hamilton, V., Baird, K., & Fenwick, J. (2020) | Y | N | Y | N | N |
| Heath, J., Aker, R., Feld, H., Singer, R. L., & Norton, J. (2019) | Y | N | N | N | Y |
| Hood, K., Cant, R., Leech, M., Baulch, J., & Gilbee, A. (2014) | N | N | N | N | Y |
| Isaacson, J. J., & Stacy, A. S. (2004) | Y | Y | Y | Y | Y |
| Jack, K., Bianchi, M., Costa, R. D. P., Grinberg, K., Harnett, G., Luiking, M.-L., Nilsson, S., & Scammell, J. M. E. (2022) | N | N | Y | N | N |
| Jones, D., McAllister, L., Dyson, R., & Lyle, D. (2018)1 | N | N | N | N | N |
| Jones, K., Blinkhorn, L. M., Schumann, S.-A., & Reddy, S. T. (2014) | N | N | N | N | Y |
| Kavannagh, J., Kearns, A., & McGarry, T. (2015) | Y | N | N | N | Y |
| Khan, T. M., & Jacob, S. A. (2015) | Y | N | Y | N | Y |
| Kjær, L. B., Nielsen, K.-J. S., Christensen, M. K., & Strand, P. (2023) | Y | N | N | N | Y |
| Lachmann, H., Ponzer, S., Johansson, U.-B., Benson, L., & Karlgren, K. (2013) | Y | N | N | N | Y |
| Leigh, J., Littlewood, L., & Lyons, G. (2019) | Y | Y | Y | Y | N |
| Lestari, E., Scherpbier, A., & Stalmeijer, R. (2020) | Y | N | N | N | Y |
| Longman, J. M., Barraclough, F., & Swain, L. S. (2020) | Y | N | Y | N | Y |
| McGettigan, P., & McKendree, J. (2015) | Y | N | N | N | Y |
| Mihaljevic, A. L., Schmidt, J., Mitzkat, A., Probst, P., Kenngott, T., Mink, J., Fink, C. A., Ballhausen, A., Chen, J., Cetin, A., Murrmann, L., Müller, G., Mahler, C., Götsch, B., & Trierweiler-Hauke, B. (2018) | Y | N | Y | N | Y |
| Mink, J., Mitzkat, A., Krug, K., Mihaljevic, A., Trierweiler-Hauke, B., Götsch, B., Wensing, M., & Mahler, C. (2021) | Y | N | Y | N | Y |
| Morphet, J., Hood, K., Cant, R., Baulch, J., Gilbee, A., & Sandry, K. (2014) | Y | N | Y | N | N |
| Musolino, G. M., & Feehan, P. (2004) | Y | N | Y | N | Y |
| Nagelkerk, J., Thompson, M. E., Bouthillier, M., Tompkins, A., Baer, L. J., Trytko, J., Booth, A., Stevens, A., & Groeneveld, K. (2018) | Y | N | N | N | N |
| Neistadt, M. E., & Cohn, E. S. (1990) | Y | N | Y | N | Y |
| Ng, E., & Hu, T. (2017) | Y | N | N | N | N |
| Nicole, M., Fairbrother, M., Nagarajan, S.V., Blackford, J., Sheepway, L., Penman, M., & McAllister, L. (2015) | Y | N | N | N | Y |
| O’Brien, D., Swann, J., & Heap, N. (2015) | Y | N | N | N | Y |
| O’Connor, A., Liston, E., & O’Donnell, M. (2019) | Y | N | Y | N | Y |
| Oosterom, N., Floren, L. C., Ten Cate, O., & Westerveld, H. E. (2019) | Y | N | Y | N | N |
| Palombaro, K. M., Dole, R. L., & Lattanzi, J. B. (2011) | Y | N | Y | N | N |
| Paparella-Pitzel, S., Anderson, E. Z., Rothpletz-Puglia, P., & Parrott, J. S. (2021) | Y | N | Y | N | Y |
| Patterson, F., Fleming, J., Marshall, K., & Ninness, N. (2017) | Y | N | Y | N | Y |
| Peterson, S. J., & Schaffer, M. J. (1999) | N | N | Y | N | Y |
| Powers, C. A., Thomson, C. C., Feuerstein, I., Cross, M., Powers, E. M., Prout, M., & Geller, A. C. (2008) | N | N | N | N | N |
| Rath, C., Tillman, F., Stickel, J., Jones, M., & Armistead, L. (2019) | Y | N | N | N | N |
| Reeves, S., Freeth, D., McCrorie, P., & Perry, D. (2002) | Y | N | Y | N | Y |
| Reime, M. H., Bell, K., Albrigtsen, B., & Beisland, E. G. (2022) | Y | N | Y | Y | Y |
| Richards, E. A., Novak, J. C., & Davis, L. V. (2009) | N | N | N | N | N |
| Rupert, D. D., Alvarez, G. V., Burdge, E. J., Nahvi, R. J., Schell, S. M., & Faustino, F. L. (2022) | N | N | N | N | N |
| Schneider, A. R., Stephens, L. A. M., Ochoa Marín, S. C., & Semenic, S. (2018) | Y | N | N | N | Y |
| Shields, N., Bruder, A., Taylor, N. F., & Angelo, T. (2013) | N | N | N | N | Y |
| Simmons, K., Klein, M., Stevens, C., & Jacobson, T. (2019) | Y | N | Y | N | Y |
| Smith, B. D., Marshall, I., Anderson, B. E., & Daniels, K. K. (2017) | N | N | Y | N | Y |
| Stuhlmiller, C. M., & Tolchard, B. (2015) | N | N | N | N | N |
| Tokolahi, E., Broman, P., Longhurst, G., Pearce, A., Cook, C., Andersen, P., & Brownie, S. (2021) | Y | N | N | N | N |
| Tsang, E. S., Cheung, C. C., & Sakakibara, T. (2016) | N | N | N | N | N |
| Tyndall, D. E., Kosko, D. A., Forbis, K. M., & Sullivan, W. B. (2020) | N | N | N | N | N |
| VanGraafeiland, B., Sloand, E., Silbert-Flagg, J., Gleason, K., & Dennison Himmelfarb, C. (2019) | N | N | Y | N | Y |
| Vijn, T., Fluit, C., Kremer, J., Faber, M., & Wollersheim, H. (2017) | N | N | N | N | N |
| Voss, H. C. (2016) | Y | N | N | N | N |
| Wilson, O. W., Broman, P., Tokolahi, E., Andersen, P., & Brownie, S. (2023) | N | N | N | N | N |
